# Repurposing neurological drugs for brain cancer therapeutics: A systematic approach to identify charged molecules for affinity-based local drug delivery systems

**DOI:** 10.1016/j.ijpharm.2025.125935

**Published:** 2025-09-15

**Authors:** Sabarni Sarker, Ben Newland

**Affiliations:** a School of Pharmacy and Pharmaceutical Sciences, Cardiff University, King Edward VII Avenue, Cardiff CF10 3NB, United Kingdom; b Department of Pharmacy, Jagannath University, Dhaka 1100, Bangladesh; c Leibniz-Institut für Polymerforschung Dresden e.V., 01069 Dresden, Germany

**Keywords:** Affinity-based drug delivery, Brain neoplasms, Drug repurposing, Local drug delivery, Glioblastoma

## Abstract

Treatment of glioblastoma (GBM) presents significant challenges due to its high degree of heterogeneity and poor prognosis. Recurrence of the malignancy is frequent even after the standard treatment, including surgery, radiotherapy, and chemotherapy with temozolomide. Drug repurposing offers a cost-effective strategy to identify new treatments, while affinity-based local delivery systems could provide controlled release of therapeutics within the tumor resection cavity. The aims of this study are two-fold. Firstly, to categorize currently available neurological therapeutics according to their charge and suitability for use with affinity-based drug delivery systems. Secondly, to systematically search and evaluate the evidence for anticancer and/or anti-glioblastoma activity for potentially being repurposed/repositioned. The ’neurology/psychiatry’ category of the Broad Institute Drug Repurposing Hub (468 compounds) was screened for chemical suitability and anticancer efficacy. Charge, lipophilicity, solubility at pH 7.4 were calculated using Chemicalize. A systematic search for the anticancer efficacy of the charged compounds was carried out via the following databases: PubMed, Scopus, Sci-Finder, Ovid via Medline, Cochrane and ClinicalTrial.gov. Among the 468 compounds, 283 were identified as charged at physiological pH. Notably, 146 charged candidates were found to have anticancer activity, of which 91 showed promising activity against at least one type of brain neoplasm. A few compounds, such as chlorpromazine, valproic acid and sertraline were investigated in clinical settings, while most were assessed through in vitro viability studies. The data complied herein should serve as a repository and starting point for future research on repurposing neurological drugs with anticancer properties via electrostatic affinity-based drug delivery systems.

## Highlights

• 468 neurological drugs were searched systematically for suitability in affinity-based delivery and efficacy in brain tumor.
• Among the charged compounds, 91 were found to have anti-brain tumor efficacy in literature.
• Findings provide a repository for neurological drugs potential for repurposing in the local delivery in brain tumor.

## Graphical abstract

**Figure:**
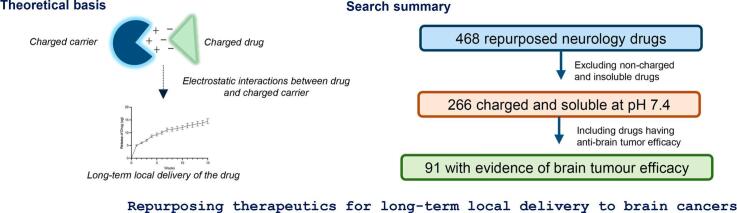

## 1 Background

Primary brain cancers are generally associated with poor survival outcome and a low quality of life, with a growing trend of global incidence and mortality (Ilic and Ilic, 2023). The therapeutic success against higher-grade brain cancers such as glioblastoma (GBM), is limited and maximal surgical resection along with temozolomide (TMZ) chemotherapy and radiotherapy is considered as the ideal intervention protocol (Rong et al., 2022, Stupp et al., 2005). However, even with the standard therapy, the median overall survival is less than 19 months, and typically only 6 % of GBM patients survive more than 5 years (Obrador et al., 2024). The main reasons behind the lack of treatment options and limited success in treatment of GBM are the degree of intra- and inter-tumor heterogeneity, the unique tumor microenvironment, tumor invasion into different regions of the brain, and direct intercellular communication by extracellular vesicles resulting in drug-resistance and angiogenesis, and subsequent recurrence of the tumor. Furthermore, physiological barriers such as the blood–brain barrier (BBB) severely limits the treatment options (Bastiancich et al., 2021b, Gao et al., 2020, Rong et al., 2022).

Using the current understanding of brain tumors, researchers have identified existing drugs of different pharmacological categories to be potentially effective (Alomari et al., 2021, Basso et al., 2018, Tan et al., 2018, You et al., 2022). Among them, neurological drugs with additional anticancer properties can be attractive candidates, because most of them can cross the BBB, and some of them might give extra therapeutic benefits in concomitant symptoms such as seizures, or depression (Abadi et al., 2022, Lyne and Yamini, 2021). However, the dose for anticancer activity of the repurposed drugs is likely to be higher than what is currently used clinically, which might be associated with toxicities in different organ systems (Schipper et al., 2022, Xia et al., 2024). Local delivery to the target site has the potential to deliver a high payload to a specific region and deliver the drugs in a controlled manner, whilst at the same time avoiding the systemic side effects and toxicities (Bastiancich et al., 2021a). This is highly relevant for brain cancer therapeutics as surgical removal of tumors is common, thus granting access for the direct delivery of drug molecules to the resection cavity (Wang et al., 2023b).

To stop recurrence of tumors, the ideal scenario for locally administered therapeutics would be a delivery system that can deliver the drug over a long time period in a controlled manner (Bastiancich et al., 2021a, Cha et al., 2022). Affinity-based interactions between the delivery system and drugs/biomolecules holds the potential for such attributes of delivery (Dogan et al., 2022, Newland et al., 2020, Teal et al., 2024). Among the non-covalent weak interactions, electrostatic affinity in particular, can be the factor controlling the release of a charged molecule from a polymer. Furthermore, the release can be tuned, based on surface area of the carrier, pH, and modification of the charges (Pakulska et al., 2016). In addition, as many polymer-based systems can be either cationic, anionic or zwitterionic in nature, charged drugs or biomolecules will have different electrostatic affinity towards these systems. For example, doxorubicin is a cationic drug with a –NH_3_ + group and electrostatically binds to a negatively charged (O-) acrylic acid block polymer (Tian et al., 2007). Later, a negatively charged amino acid-based gelatin and hydroxyethyl methacrylate-based hydrogel showed sustained release of doxorubicin over 5 days with the release profile being dependent on pH (Çetin et al., 2017). Furthermore, in a study, the electrostatic attraction between doxorubicin and polyethylene glycol-heparin cryogels resulted in 42 days of controlled release of the drug (Newland et al., 2020). Another study suggested pH and temperature-dependent controlled release of diclofenac (with O- group) from N,N-dimethylaminoethyl methacrylate, and acrylamide copolymer-based hydrogels (containing NH + groups) (Dragan and Cocarta, 2016). Zwitterionic systems, such as injectable sulfobetaine methacrylate cryogels, have been described for long-term delivery of doxorubicin for the treatment of breast cancer (Jing et al., 2023).

In summary, several neuroactive drugs feature charge(s) and may be good candidates for repurposing with affinity-based drug delivery systems. Furthermore, there is mounting evidence of some of these having anti-tumor or anti-glioblastoma properties. To the best of our knowledge, there is no systematic representation of neuroactive drugs based both on their physical charge and their anti-cancer or anti-glioblastoma properties. The aim of this study was to screen a drug repurposing database for neuroactive drugs potentially suitable for delivery via affinity-based delivery systems and systematically search the literature to identify evidence of efficacy against glioblastoma or other cancers.

## 2 Methodology

### 2.1 Screening for drugs chemically suitable for affinity-based delivery systems

The Broad Institute Drug Repurposing Hub (https://clue.io/repurposing) was used to retrieve a list of neuroactive therapeutics. This database contains a list of 6798 preclinical, clinical, and withdrawn compounds. To narrow down the drugs based on their existing medical use, the 'neurology/psychiatry' box was selected from the disease area column, and 468 initial compounds were included for screening (updated: 07 October 2024).

Chemicalize software (https://chemicalize.com/) was then consulted to categorize the 468 drugs based on their charge and solubility at pH 7.4. Of these, compounds in the ‘neurology/psychiatry’ disease area, also having charges (+/-/both) and an aqueous solubility profile of at least 0.01 mg/ml, were subsequently included for further literature screening for their anticancer activity for brain and other neoplasms. From the same tool, molecular weight and logD (a measurement of lipophilicity) at pH 7.4 was also noted as additional information.

### 2.2 Screening for drugs with anticancer and anti-GBM activity

**Fig. 1:**
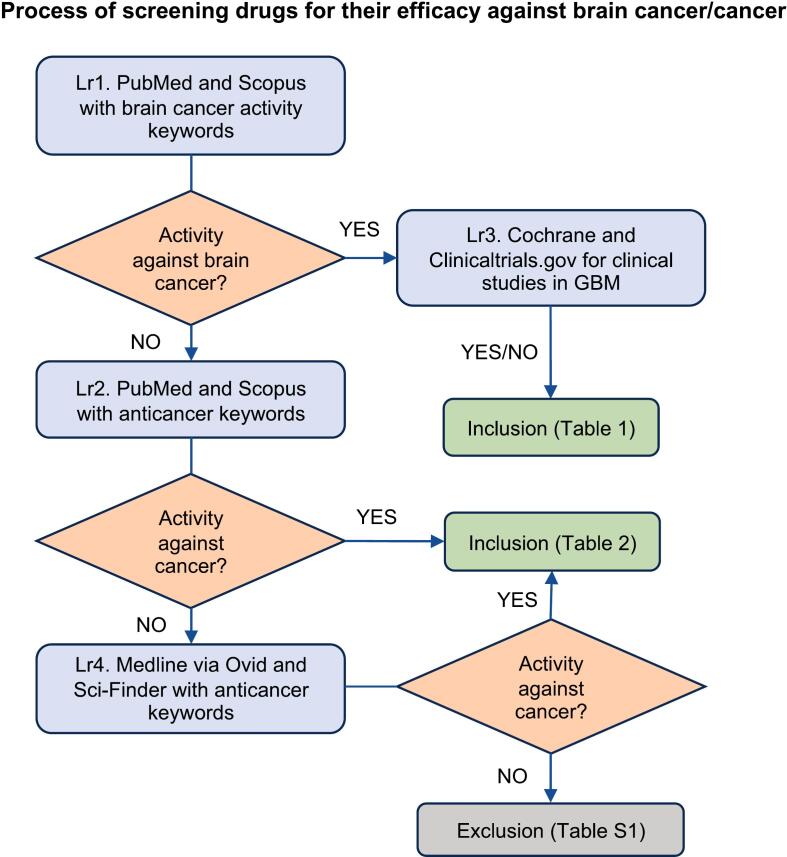
Flow chart explaining the screening of a charged drug for its anticancer activity. Layer (Lr) e.g., Lr1, Lr2, Lr3 and Lr4 indicate different search layers. Those included for anti-brain tumor activity are displayed in Table 1, those included for any anticancer activity (green box) are combined in Table 2, while drugs with no evidence of anticancer efficacy are put in the **Table S1.**

6 databases (PubMed, Scopus, Sci-finder, Medline via Ovid, Cochrane, and Clinicaltrials.gov) were systematically consulted with specific terms. In general, the search strategy was multi-layered and designed as follows (summarized in Fig. 1).

*Layer 1*: PubMed and Scopus were the primary databases consulted for any evidence of an effect of the compound in GBM and other forms of brain neoplasm. The following search term were used:

PubMed: ((drug name[Title/Abstract]) AND ((“brain tumo*”[Title/Abstract]) OR (“brain cancer”[Title/Abstract]) OR glioblastoma[Title/Abstract] OR glioma[Title/Abstract] OR astrocytoma[Title/Abstract] OR oligodendroglioma[title/abstract] OR medulloblastoma[title/abstract] OR meningioma[title/abstract] OR ependymoma[title/abstract] or neuroblastoma[title/abstract] OR “brain neoplasm*”)).

Scopus: TITLE-ABS (drug name) AND TITLE-ABS (“brain tumo*” OR “brain cancer” OR glioblastoma OR glioma OR astrocytoma OR oligodendroglioma OR medulloblastoma OR meningioma OR ependymoma OR neuroblastoma OR “brain neoplasm*”).

To note, for the 'drug name', the MeSH (Medical Subject Heading) term of the compound was searched using the Ovid via Medline tool (although this tool was not a main part of this search layer), and it was incorporated in the string. In addition, the terms for specific forms of brain cancer were selected based on their prevalence, while the MeSH term 'brain neoplasm*' was also included (Chien et al., 2016, Miller et al., 2021).

*Layer 2*: If there were no relevant results from layer 1, PubMed and Scopus were further consulted for evidence of general anticancer activity of the therapeutics, and the search strategy was as follows:

PubMed: ((*drug name*[Title/Abstract]) AND (Cancer[Title/Abstract] OR tumo*[Title/Abstract] OR sarcoma[Title/Abstract] OR carcinoma[Title/Abstract] OR neoplasm*[Title/Abstract])).

Scopus: TITLE (*drug name*) AND TITLE-ABS (cancer OR tumo* OR sarcoma OR carcinoma OR neoplasm).

*Layer 3*: If there were already relevant results from layer 1, Cochrane and Clinicaltrials.gov were searched additionally for published and unpublished clinical trials of the drugs. For Cochrane, the strategy was as follows.

Cochrane: title abstract keyword: (*drug name*) AND (“brain tumor” OR “brain cancer” OR glioblastoma OR glioma OR astrocytoma OR oligodendroglioma OR medulloblastoma OR meningioma OR ependymoma).

For Clinicaltrials.gov, the advanced search methods were used to input the intervention (drug name) and disease name as (brain cancer), (glioblastoma), or (glioma) separately.

*Layer 4*: This is a layer created for further validation of the systematic search. It was conducted for drugs with no relevant results from layer 2. Two separate database Medline via Ovid and Sci-finder were consulted for this step. As PubMed (used in layer 1) extracts the abstracts from the same repository (MEDLINE) as Ovid, a similar but slightly different search method was formulated for this validation step (memantine as an example of MeSH term for a drug):

1 Glioblastoma/ or Central Nervous System Neoplasms/ or Brain Neoplasms/ or Astrocytoma/ (157934).

2 Neoplasms/ (535135).

3 Antineoplastic Agents/ (336119).

4 Cell Line, Tumor/ (398828).

5 1 or 2 or 3 or 4 (1254201).

6 Memantine.mp. (4751).

7 5 and 6 (128).

Additionally in this step, Sci-Finder helped to search for relevant patents that were not found in other databases. This database has a smart advanced search engine, therefore following search term were used: “abstracts, keywords: (drug AND tumor)”, “abstracts, keywords: (drug AND anticancer)”, and “abstracts, keywords: (drug) AND concept: (anticancer)”.

### 2.3 Validity assessment and inclusion criteria

From the results with specific search terms from each database, review articles, short communications, and conference papers were removed from the results. Then, the duplicate results were removed. For searching the drugs acting on brain cancers, the title and the abstract are thoroughly checked for relevance based on following metrics:

• Clear indication of an in vitro, in vivo or clinical study with certain outcome measures, including but not limited to viability, apoptosis, migration, proliferation, tumor volume, and overall survival.
• Clear indication of use of a brain cancer cell line even if the objective is different, such as the biocompatibility studies of certain compounds with neuroblastoma cell lines.

After the abstract and title was screened, full text was read for the selected papers. After that, drugs (rather than the papers) were selected based on the following inclusion criteria:

• One clinical study against any form of brain tumor.
• Positive therapeutic outcome in in vivo xenograft or other models in terms of survival or tumor growth.
• Positive therapeutic outcome in in vitro brain cancer cell line in terms of inhibition of cell viability, migration, proliferation, invasion, metabolism, mitochondrial activity, and cell cycle.
• In silico prediction of prevention with certain pathways of brain cancer, only if the drug has a positive therapeutic outcome against at least one in vitro cell line models of other forms of cancer.

### 2.4 Looking for additional properties for screened drugs

Using the Simplified Molecular Input Line Entry System (SMILES) of the compound, the theoretical BBB permeation profile was retrieved from Swiss ADME (https://www.swissadme.ch/) and pkCSM, a computational tool for pharmacokinetics calculation of small molecule (https://biosig.unimelb.edu.au/pkcsm/prediction). LogBB, the logarithmic value of concentration of the drug in the cerebrospinal fluid (CSF) divided by the concentration of the drug in the blood can be used to predict the BBB permeability of a drug, was calculated from pkCSM tool (Pires et al., 2015). The values were added in a column of the Table 1 and Table 2. A positive logBB might indicate the possibility of penetration of the BBB by the drug (Carpenter et al., 2014).

**Table 1:** Charged drugs having evidence of efficacy as brain cancer therapeutics.

**Drug name**	**Class by mechanism**	**MW,** **charge^a^,** **solubility^b^**	**Evidence of anticancer action in brain neoplasms**	**LogBB**
(R)-(−)-apomorphine	DRA	267.328 + 1.65	Cell viability inhibition in U3046MG cells (Pinheiro et al., 2017); growth inhibitory effects on T98G, A172, U251MG, U57MG, and primary GBM cell lines (Lee et al., 2016b).	0.344
Amantadine	GluRB	151.253 + 151.25	In vivo improvement of survival of BALB/c nude mice with U251 xenograft with dose 50 mg/kg/day (Luo et al., 2024); antiproliferative activity on cell lines A172 and U87MG (Kasemsuk et al., 2019).	0.828
Amitriptyline	SNRI, TCA	277.411 + 0.53	Mitochondrial activity inhibition in T98G and U87 cells (Bielecka-Wajdman et al., 2017); cytotoxic to C6 (Slamon et al., 2001); astrocytoma 1321N1n (Slamon and Pentreath, 1998), IPSB-18 and GBM-derived CLOM 15 cells (Higgins and Pelkington, 2010).	0.972
Amoxapine	NRI	313.79 + 0.17	Lysosomal membrane damage in patient derived GBM cell line TGS04 (Jing et al., 2022).	0.312
Aripiprazole	SRA, SRB	448.39 + 0.33	Cytotoxic effects in U251 and LN428 cells (Kim et al., 2018); but no cytotoxic effects on C6 glioma cells were reported (Baek et al., 2015).	−0.052
Asenapine	Atypical antipsychotic	285.77 + 0.05	A patent claimed in vitro cytotoxic effect on pediatric LAN-1 and U87 cells (Soto Cerrato et al., 2022).	0.664
Aspirin	COX-I	180.159 (−) 255.11	Inhibition of proliferation of patient derived GBM cells (Pozzoli et al., 2019a) and SK-N-SH (Pozzoli et al., 2019b); prevention of angiogenesis on human primary GBM–endothelial cells (Navone et al., 2018) and C6 glioma cells (Qin et al., 2016); growth inhibition of rat glioma G2 (Aas et al., 1995), apoptosis on U87 (Chang et al., 2020, Huang et al., 2013, Lan et al., 2011), neuroglioma H4 (Chang et al., 2020), U251 (Huang et al., 2013), A-172 (Kim et al., 2009, Lan et al., 2011), GBM T98G (Amin et al., 2003), cell death in GBM U87 in tumor-bearing BALB/c nude mice (Chang et al., 2020), tumor size diminished in rat glioma C6 xenograft at low-dose (Arrieta et al., 2001), Phase I clinical trial (O'Rawe et al., 2022).	−0.34
Atorvastatin	Lipid lowering agent, HMGCR inhibitor	558.65 (−) 0.06	Cell-specific cytotoxicity on A172 cells (Oliveira et al., 2018); concentration dependent toxicity on 2D GBM6840 cells, 3D model of the mouse GBM CT-2A cells and mouse brain tumor-initiating bRiTS-G2 cells (Luebtow et al., 2020); treatment of C6 glioma induced rat model (Goodarzi et al., 2020); cytotoxicity on U87 (Bayat et al., 2016, Nooshabadi et al., 2020, Yi et al., 2013), U251 (Cui et al., 2022); antiangiogenic effects on U87 cells (Valipour et al., 2022, Yi et al., 2013).	−0.731
Baclofen	Benzodiazepine receptor agonist	213.66 (−) 4.28	Apoptosis in U251 and U251 induced xenograft in mice (Zhu et al., 2021).	−0.018
Baicalin	Beta glucuronidase inhibitor	446.364 (−) 446.63	Cytotoxicity on U87 (Ibrahim et al., 2022, Paul-Samojedny et al., 2023, Zhu et al., 2018) and U251 cells (Zhu et al., 2018); inhibitory effect on rat C6 glioma cells (Hu et al., 2013).	−1.716
Biperiden	AchRB	311.469 + 15.71	Antiproliferative activity on A172, LN229, SF268 and SK-N-SH cell lines (Doello et al., 2022).	0.547
Blonanserin	DRB, SRB	367.512 + 0.02	Cytotoxic effect on U251 and T98G cells (Tsuchiya et al., 2023).	0.403
Bremelanotide	Melanocortin agonist	1491.11 + 453.46	Reduction of cell viability in U87, GS-Y01 and GS-Y03 cells (Suzuki et al., 2024).	−3.948
Brexpiprazole	DRA (partial)	433.57 + 0.02	Cytotoxic activity to glioblastoma stem cell line GS-Y03 (Suzuki et al., 2019b); chemo-sensitizing property to an anti-EGFR agent osimertinib against glioma stem cells (Suzuki et al., 2019a).	0.105
Bupivacaine	NaChB	288.435 + 0.22	Apoptotic effect in SH-SY5Y, and mouse Neuro2a cells (Ji et al., 2020, Liang et al., 2016, Perez-Castro et al., 2009).	0.321
Chlorogenic acid	Antioxidant	354.311 (−) 446.98	Phase II clinical trial for glioma treatment (Li et al., 2024b); cell differentiation in Be(2)-M17, SH-SY5Y cells (You et al., 2023), antitumor effects on M059J, U87MG cells (Huang et al., 2019), inhibition by macrophage polarization in U87MG lines, and mouse G422 glioma cell xenograft (Xue et al., 2017), Inhibition of proliferation of U373 cells (Zhou et al., 2021), targeted delivery of liposome formulation to prevent G422 glioma cell growth (Ye et al., 2020).	−1.423
Chloroprocaine	NaChB	270.76 + 12.53	Cytotoxic effect on SH-SY5Y and other NB cells (Perez-Castro et al., 2009, Seravalli and Lear, 1987).	0.066
Chlorpromazine	DRB	318.86 + 0.29	Anticancer effect on T98G, U-251 MG and U-87 MG cells (Matteoni et al., 2021a, Matteoni et al., 2021b, Shin et al., 2013).	0.861
Chlorprothixene	DRB	315.86 + 0.17	Growth inhibition in the DOAY medulloblastoma cell lines (Kurita et al., 2018).	0.959
Cinacalcet	CaChA	357.42 + 0.09	Apoptosis in neuroblastoma cells LA-N-1, LA1-55n, SH-SY5Y, SK-N-JD, SK-N-LP, LA1-5 s, SK-N-AS and IMR5 (Goncalves-Alves et al., 2022, Rodriguez-Hernandez et al., 2016).	0.801
Cinnarizine	CaChB	368.524 + 0.03	Reduction of viability of NB 69 cells (Mena et al., 1995).	1.227
Citalopram	SSRI	324.399 + 44.16	Cytotoxicity on C6 glioma cells (Martínez-Díaz et al., 2020); selective cytotoxicity on rat B104, human SH-SY5Y, IMR32, Kelly human NB cells in comparison to human Schwann cells (Sakka et al., 2017).	−0.271
Clomipramine	SRI	314.86 + 0.55	Inhibition of cellular respiration of the astrocytoma cell IPSB-18 (Higgins and Pelkington, 2010); cytotoxicity on SH-SY5Y (Ayla et al., 2014), SK-MEL28 melanoma cell line, and melanoma primary cell culture (Parker et al., 2012).	0.915
Clozapine	DRB, SRB	326.83 + 0.23	Affecting cell viability significantly in U87MG (Karbownik et al., 2016); neurotoxic to SK-N-SH (Gasso et al., 2023, Sharp et al., 2013); inhibition of cell cycle progression of U87MG (Shin et al., 2006).	0.731
Dabigatran	Thrombin inhibitor	471.521 (+ −) 0.02	Growth and cell cycle progression antagonism in U87‐MG cells (Vianello et al., 2016).	−1.034
Desipramine	TCA	266.388 + 27.04	Cytotoxic effects by autophagy induction in C6 glioma cells (Ma et al., 2013).	0.758
Diclofenac	COX-I	296.15 (−) 36.9	Antiproliferative effects on U87 cells (Sareddy et al., 2013); cytotoxicity on C6 glioma cells (Lopes et al., 2021), U87MG and A172 cell lines (Leidgens et al., 2015); cell cycle and growth inhibition of murine GL261 glioma cells and tumor size reduction in xenograft model (Chirasani et al., 2012); apoptosis in SH-SY5Y (Johnsen et al., 2004, Ruocco et al., 2010); tumor growth inhibition in SH-SY5Y xenograft (Johnsen et al., 2004).	0.044
Donepezil	Acetylcholinesterase inhibitor	379.5 + 2.33	Antitumor effect against Hs683, T98G and U373 cells (Mégalizzi et al., 2009).	0.479
Dopamine	DRA	153.181 + 7018.6	Cytotoxicity reported for various cancer cell lines, including some of the brain cancer cells (Clement et al., 2002).	−0.337
Doxepin	HRB	279.383 + 2.19	Inhibition of cellular respiration of anaplastic astrocytoma-derived IPSB-18 cells (Higgins and Pelkington, 2010).	0.81
Duloxetine	SNRI	297.42 + 0.32	Reducing glioma tumor growth in vivo (xenograft) by blocking S100B (Gao et al., 2018).	0.527
Escitalopram	SSRI	324.399 + 44.16	Selective cytotoxicity to B104 rat neuroblastoma cell line, human SH-SY5Y, IMR32, Kelly human neuroblastoma cell lines in comparison to human Schwann cells (Sakka et al., 2017), rat C6 glioma cells (Dikmen et al., 2011), apoptosis in U87MG, autophagy in GBM8401 cells (Chen et al., 2018).	−0.271
Fasudil	Rho associated kinase inhibitor	291.37 + 7.14	Inhibition of growth and progression of GBM cell lines U251, T98G and eGFP-T98G (Deng et al., 2010).	−0.147
Fingolimod	Sphingosine 1-phosphate receptor agonist	307.478 + 0.19	Cytotoxic for SF-268, NB SK-N-SH and SH-SY5Y, A172, LN-229, G28 and U87 cells and human medulloblastoma cells D283 and DAOY (Doello et al., 2022, Kolodziej et al., 2020, Perla et al., 2020, Rank et al., 2022).	−0.329
Fluoxetine	SSRI	309.332 + 2.66	Selective cytotoxicity to U87 and GBM8401 cells in comparison to rat astrocytes, in vivo suppression of tumor growth in U87 xenografts (Liu et al., 2015); one study found that it kills GBM cells by disrupting sphingomyelin metabolism by inhibition of SMPD1 (Bi et al., 2021).	0.505
Fluphenazine	DRA	437.53 + 0.45	Cell viability reduction of NB cell lines CLBGA, IMR32, NGP, SKNBE, SKNSH (De Preter et al., 2009), and C6 rat glioma cells (Gil-Ad et al., 2004).	0.594
Fluspirilene	DRB	475.584 + 0.01	Decreased cell viability in three glioma stem cell lines TGS01, TGS04, and KGS01; inhibition of STAT3 activity and inhibition of proliferation of U251, SNB19, T98, and U87 cell lines (Dong et al., 2017), cytotoxic for U87MG and U251 cells (Varalda et al., 2020).	0.16
Fluvoxamine	SSRI	318.34 + 8.53	Inhibition of cell migration and invasion of A172, U87MG, and U251MG cells (Hayashi et al., 2016).	−0.312
Gabapentin	CaChB	171.24 (+ −) 12.84	One study reported no cytotoxic effect on C6 glioma (Güneri et al., 2022), while another one reported dose dependent decrease in cell viability (Kaur et al., 2016).	−0.186
Glucosamine	Glycosylated protein precursor	179.172 + 1863.13	Cytotoxic activity on SH-SY5Y cells (Sahin et al., 2022); growth inhibitory effects on C6 glioma cells (Friedman and Skehan, 1980).	−1.07
Haloperidol	DRB	375.87 + 0.10	Antitumor effects on T98G and U373 cells (Mégalizzi et al., 2009), cytotoxic for astrocytoma cells SF-268, NB SK-N-SH, GBM lines A172, LN-229 (Doello et al., 2022), U87, U251 and T98 cells (Papadopoulos et al., 2020).	−0.104
Ibuprofen	COX-I	206.285 (−) 20.94	Cytotoxic activity on U-251 (Pedro-Hernández et al., 2020), A172, U87MG, U138MG, U251MG, and T98G cells (Ferreira et al., 2021).	0.302
Imipramine	SNRI	280.415 + 2.56	Cytotoxicity on U87 (Jeon et al., 2011), LN-229, LN-71 and LN-443 cells (Shchors et al., 2015), apoptosis on U-87 MG and GBM8401 cells (Hsu et al., 2020).	0.937
Levomepromazine	DRB	328.47 + 0.95	Cytotoxic for SF-268, SK-N-SH, A172 and LN-229 cells (Doello et al., 2022).	1.027
Lidocaine	LA	234.343 + 1.77	Inhibition of proliferation (Leng et al., 2017) and autophagy induction (Izdebska et al., 2019) in C6 glioma cells; inhibition of progression U251MG and T98G (Wen et al., 2021), apoptosis in SH-SY5Y cells (Li and Han, 2015).	0.28
Maprotiline	TCA	277.411 + 176.22	Phase I clinical trial of GBM with TMZ, which is withdrawn (Petrosyan et al., 2022), in silico prediction (Lee et al., 2016b), also antitumor effect in melanoma (Liang et al., 2024).	0.896
Meclizine	Constitutive androstane receptor agonist	390.96 + 0.01	Reduced cell viability in patient derived GBM cells (Sandoval et al., 2020).	1.217
Meclofenamic acid	COX-I	296.15 (−) 7.18	Disruption of cell–cell communication (cellular tethering and functional networks) in primary GBM cells (Schneider et al., 2021).	0.256
Memantine	GluRB	179.307 + 179.31	Decrease of cell viability of T98G and U87-MG cell lines (Albayrak et al., 2021), induction of autophagy and inhibition of proliferation of T-98G and U-251MG cells (Yoon et al., 2017).	0.603
Mitoxantrone	(Cancer and multiple sclerosis)	492.654 (+ +) 444.49	Mainly anti-prostate and breast cancer agent, clinically, but also multiple studies for anti-glioma activity.	−2.183
Monomethyl fumarate	Nrf2 activators	129.092 (−) 7496.73	Reduced cell viability in SH-SY5Y cells in a study with modified formulation (Kumar et al., 2018), dimethyl fumarate, its precursor form also has an effect on mouse glioma GL261, human GBM A172 cells (Ghods et al., 2013).	−0.383
Nicotine	AchRA	162.236 + 2231.28	Dose dependent cytotoxicity in glioma KG-1-C, GBS-1 and T98G cells (Yamamura et al., 1998). Cell death in SH-SY5Y cell lines (Li et al., 2024a).	0.208
Niflumic acid	COX-I	282.222 (−) 6.83	Effect on proliferation, migration and invasion of glioma U87 cells (Cui et al., 2020).	0.023
Olanzapine	DRB	312.44 + 0.21	Autophagy induction in T98, LN229, and U87 cells (Zhu et al., 2019); inhibition of proliferation of glioma stem-like cells (Guo et al., 2015); exerts neuroprotective effects on SH-SY5Y cells (Lee et al., 2010).	0.376
Paliperidone	DRB	426.492 + 56.07	Reduced tumor volume and increased survival in mouse xenograft with GBM line ALTS1C1 (Liu et al., 2021a), neuroprotective activity in SK-N-SH cells (Gassó et al., 2012).	−0.753
Parecoxib	COX-I	370.42 (−) 0.24	Effect on growth, proliferation and invasion of U251 and U343 cells at 100-200uM doses (Li et al., 2017a).	−0.612
Paroxetine	SSRI	329.371 + 2.98	Selectively effective in patient derived GBM cells (Lee et al., 2024).	0.093
Perphenazine	DRB	403.97, + 0.71	Antiproliferative effects (Tzadok et al., 2010), effects on migration and invasion in U87 cells (Otreba et al., 2022); apoptosis in C6 and SH-SY5Y cells (Gil-Ad et al., 2004), and synergistic effect with TMZ in patient derived tumor spheres (Hong et al., 2025).	0.619
Pimavanserin	Serotonin receptor inverse agonist	427.564, + 0.05	Inhibition of growth of U87 cells, IC50: 1.46–8.07 μM (Liu et al., 2021b).	0.043
Pimozide	DRB	461.557, + 0.01	Selective antitumor activity on patient derived GBM cells (Lee et al., 2024); apoptosis on U87MG, DAOY, GBM 28, and U251MG cells; IC50 12–16 µM (Ranjan et al., 2020), antiproliferative effects on G6 cell lines; IC50 8 µM (Bertolesi et al., 2002).	0.249
Prilocaine	LA	220.316 + 12.40	LD50 4.32 ± 0.39 in SH-SY5Y cells (Malet et al., 2015); reduced growth (size) of U251 tumor in vivo (Fan et al., 2021); apoptosis on NB2a cells (Mete et al., 2015).	0.215
Procaine	LA	236.315 + 60.37	Reduced growth (size) of U251 tumor in vivo (xenograft) (Fan et al., 2021); apoptosis on NB2a cells (Mete et al., 2015), LD50 39 mM in SH-SY5Y cells (Perez-Castro et al., 2009).	0.067
Propranolol	Adrenergic antagonist	259.349 + 3.86	In one study with 15 patient derived NB cell lines, propranolol had IC50 value 114–218 μM (Pantziarka et al., 2016).	0.258
Protriptyline	TCA	263.384 + 151.31	In silico study predicts it can be effective in GBM, binding to targets such as PARP1, PARP2, PRG, RBP1 to disrupt DNA repair pathways (Lin et al., 2022); and also Ca independent cell death in osteosarcoma (Su et al., 2016), and prostate cancer cells (Chang et al., 2015).	0.818
Quetiapine	DRB, SRB	383.51 + 0.07	Antitumor effect in LN-308, LN-229 and several patient derived cell lines (Lee et al., 2024); 50 % of cell viability in GSC at 50 µM doses (Wang et al., 2017a).	0.028
Rasagiline	MAO-I	171.243 + 1.71	High relative cytotoxicity score for LN-308 cells (Lee et al., 2024); on the contrary, a neuroprotective effect on U118MG by increasing BDNF and GDNF (Inaba-Hasegawa et al., 2017).	0.624
Risperidone	DRB SRB	410.493 + 21.96	Cytotoxic to LN-308 cells (Lee et al., 2024); decreased induced PDL1 expression in U251 and mouse ALTS1C1 cell lines (Liu et al., 2021a); synergistically improved effect with TMZ in U251 and A172 cells (Liu et al., 2019).	−0.064
Rivastigmine	Cholinesterase inhibitor	250.342 + 65.50	Although no significant effect, it seems to give a positive score for relative inhibition in some GBM cells, but no considerable evidence, otherwise (Lee et al., 2024).	0.508
Ropivacaine	LA	274.408 + 0.49	Inhibition of proliferation and migration on T98G and LN229 cells (Liu et al., 2020a); LD50 13.43 ± 0.61 mm for SH-SY5Y cells (Malet et al., 2015).	0.333
Rutin	Antioxidant	610.521 (−) 40.53	Decrease viability of human GL-15 GBM cells (Santos et al., 2015, Santos et al., 2011); selectively (compared to microglia) decrease viability of GL-15 at 30 µm dose (Lima et al., 2024); conversely failed to cause cell death in U87MG, C6, and U138 cells (Gentile et al., 2015).	−2.08
Safinamide	MAO-I	302.349 + 0.28	Approximately 40–50 % reduction of metabolic activity relative to control in SH-SY5Y cell lines (Knez et al., 2022).	−0.26
Salicylic acid	COX-I	138.122 (−) 1173.36	Inhibition of the growth of rat RG 2 cells (Aas et al., 1995).	−0.313
Sertraline	SSRI	306.23 + 0.64	One of the compounds in a clinical trial of 9 repurposed therapeutics with TMZ (Halatsch et al., 2021). Growth inhibition in DOAY cells (Kurita et al., 2018).	0.656
Spiperone	DRB	395.478 + 0.04	In silico prediction for interacting with GBM protein VPS11, BTG2, AP1S2 (Lee et al., 2024).	0.11
Tacrine	Acetylcholinesterase inhibitor	198.269 + 15.10	At 1 μM, reduction of mitosis and protein synthesis in murine N2A (NB) cells (Zatta et al., 1995).	0.196
Tetracaine	LA	264.369 + 6.23	In a study with rat NB2a cells, it showed higher cell viability and apoptotic potency than other local anesthetics (Mete et al., 2015).	0.272
Tiagabine	GABA uptake inhibitor, antiepileptic	375.55 (+ −) 0.03	Only 10 % growth inhibition in T98 and U87 cell lines at 0.8 µg/ml (Lee et al., 2016a).	0.201
Ticlopidine	Purinergic receptor antagonist,	263.78 + 0.29	Dose-dependent apoptotic effect on U87 cells (Chen et al., 1997).	0.899
Tolfenamic acid	COX-I	261.71 (−) 27.46	Growth reduction in NB cell lines of SH-SY5Y, CHLA90, LA1 55n, SHEP, Be2c, CMP 13Y, and SMS KCNR (Eslin et al., 2013b); reduced growth of medulloblastoma cell line-induced tumor (Eslin et al., 2013a).	0.276
Tranylcypromine	MAO-I	133.194 + 125.37	Reduction of invasion of U87 MG and 11ST patient-derived cell lines (Sachkova et al., 2019).	0.083
Trifluoperazine	DRB	407.5 + 0.27	Reduction of tumor volume and improved survival of C57BL/6 mice implanted with GL261-Luc or GL261-StrawRed murine GBM cells (Bhat et al., 2020); significant relative activity in four GBM cell lines (Lee et al., 2024).	0.875
Triflupromazine	DRB	352.42 + 0.17	In a study, in silico prediction was validated with significant relative activity in four GBM cell lines (Lee et al., 2024).	0.821
Valproic acid	Anti-epileptic	144.214 (−) 99.10	40 % growth inhibition in T98 and U87 cells in 500ug/ml (Lee et al., 2016a); antimitotic effects on NB cell lines (Güneri et al., 2022).	0.27
Varenicline	AchRA	211.268 + 2983.08	Significant relative effect in two patient derived cell lines and LN-308 cells (Lee et al., 2024).	0.526
Vilazodone	SRI	441.535 + 0.04	Significant cytotoxicity on ZH-161, ZH-562, LN-229, LN-308 cells (Lee et al., 2024).	−0.477
Vortioxetine	SRB/SRA	298.45 + 0.10	Potent AP-1 inhibitor and cytotoxic against ZH-161, ZH-562, LN-229, LN-308 cells and patient derived cell lines (Lee et al., 2024), and also inhibition of viability in HGC27, AGS​ gastric carcinoma cell lines (Li et al., 2023, Lv et al., 2020).	0.884
Ziprasidone	DRB/SRB	412.94 + 0.11	Only one patient derived GBM cell line affected in a study (Lee et al., 2024); and also cell death inducing in breast carcinoma (Sahu et al., 2023), reducing proliferation of pancreatic adenocarcinoma (Yang et al., 2022).	0.082
Zolmitriptan	Selective SRA	287.363 + 194.72	Only one patient derived GBM cell line affected in a study (Lee et al., 2024); and also Apoptosis indication in hepatocellular carcinoma rat models (Maurya et al., 2019).	−0.07
Zotepine	DRB, SRB	331.86 + 0.03	Cytotoxic against ZH-161, ZH-562, LN-229, LN-308 cells and patient derived cell lines (Lee et al., 2024).	0.736
Zuclopenthixol	DRB	400.97 + 0.22	In vivo growth reduction of intracranial tumor xenograft for HER2 + breast cancer cells (Faure et al., 2021).	0.621

MW, molecular weight; a, charge at pH 7.4; b, solubility at pH 7.4 (mg/ml); DRA, Dopamine receptor agonist; DRB, Dopamine receptor antagonist; SRA, serotonin receptor agonist; SRB, serotonin receptor antagonist; NRI, norepinephrine reuptake inhibitor; SNRI, Serotonin and NE reuptake inhibitor; NE, norepinephrine; GluRB, Glutamate receptor antagonist; AchRA, acetylcholine receptor agonist; AchRB, acetylcholine receptor antagonist; NaChB, Sodium channel blocker; CaChA, calcium channel activator/agonist; CaChB, calcium channel blocker; SSRI, selective serotonin reuptake inhibitor; HRB, histamine receptor antagonist, TCA, tricyclic antidepressant; LA, local anesthetics; HMGCR, 3-hydroxy-3-methylglutaryl-CoA reductase; BBB, blood brain barrier; COX-I, cyclooxygenase inhibitor; MAO-I, monoamine oxidase inhibitor; GBM, Glioblastoma, NB, neuroblastoma; IC50, half-maximal inhibitory concentration; BDNF, brain derived neurotrophic factor; GDNF, glial cell line derived neurotrophic factor.

**Table 2:** Drugs without published literature showing anti-brain tumor activity but having evidence of efficacy against other cancers.

**Drug name**	**Class by mechanism**	**MW,** **charge^a^,** **solubility^b^**	**Evidence of anticancer action**	**LogBB**
Ademetionine	Phosphodiesterase inhibitor	399.45 (+ + −) ∼10	Antiproliferative effects on oral, laryngeal, liver, colon, ovarian, breast cancer cells (Ilisso et al., 2018, Lu and Mato, 2008, Mosca et al., 2020, Mosca et al., 2022, Wang et al., 2017b).	−1.669
Atosiban	Oxytocin receptor antagonist	994.19 + 54.80	Decreasing tumor volume and weight in mice models of breast cancer (Khori et al., 2018); synergistic cytotoxic effect with 5-FU on colorectal cancer cells HCT-116 and HCT-8 (Wang et al., 2022).	−2.208
Benserazide	DOPA decarboxylase inhibitor	257.246 + 384.69	Effective against melanoma and colon cancer cell lines (Druzhyna et al., 2016, Laila et al., 2020, Li et al., 2017b, Zhou et al., 2020); in silico screening, effect observed on HCT116 colon cancer cells (Druzhyna et al., 2016).	−1.381
Benztropine mesylate	AchRB	307.437 + 7.93	Inhibitory effects on mouse (LuM1) and human-derived colon cancer cells (Sogawa et al., 2020), anticancer effect in breast cancer stem cells and breast cancer cell line MCF-7 (Costa et al., 2021, Cui et al., 2017).	0.706
Betahistine	HRA, HRB	136.198 + 15656.76	Cytotoxic effects on human lung adenocarcinoma A549 cell lines (Kepekçi et al., 2021).	0.104
Bupropion	Dopamine reuptake inhibitor	239.74 + 0.58	There is one patent claiming an anti-migration effect on TNBC cell line MDA-MB-231 (Yang et al., 2020).	0.258
Carbidopa	Aromatic L-amino acid decarboxylase inhibitor	226.232 (+ −) 34.38	Cytotoxic for pancreatic cancer, melanoma, ER-positive breast cancer and prostate cancer cell lines (Chen et al., 2020, Chen et al., 2022, Duarte et al., 2019, Korac et al., 2022).	−1.211
Cholic acid	Bile acid	408.579 (−) 168.49	One in silico study suggests that it can act as anticancer drug as a sphingosine kinase 1 inhibitor (Shakeel et al., 2023).	−0.679
Citicoline	Glutathione transferase stimulant	488.327 (+ − − −) ∼97	One patent showed its effectiveness in combination with another drug in the treatment of ovarian cancer (Qian, 2016).	−2.392
Clonidine	Adrenergic receptor agonist	230.09 + 1.34	Prevention of osteolytic bone metastasis (King, 2012).	0.335
Cytisine	AchRA	190.246 + 3044.20	Growth inhibition of lung cancer cells (Xu et al., 2020), Cytotoxicity in human HCC cells (Yu et al., 2017), HepG2 cells (Yu et al., 2018).	−0.17
Dimercaptosuccinic acid (DMSA)	Heavy metal chelating agent	182.21 (−) 1210.22	A study showed that it can be selectively cytotoxic to breast cancer MCF-7, cervical cancer HeLa and hepatoblastoma HepG2 cells while in conjugation with FePt–Au hybrid nanoparticles (Wang et al., 2016).	−1.212
Diphenhydramine	HRB	255.361 + 10.92	Cytotoxic against pancreatic cancer PANC-1 cell line (Zhang et al., 2021).	0.794
Entacapone	Catechol O methyltransferase inhibitor	305.29 (+ − −) 9.05	Cytotoxic to esophageal cell lines (Ramedani et al., 2023). In a study entacapone did not affect the viability of SH-SY5Y neuroblastoma cells (Korlipara et al., 2004).	−1.041
Flupentixol	DRB	434.52 + 0.13	Cytotoxicity on lung cancer cell lines (Dong et al., 2019).	0.597
Fosfosal	Phosphodiesterase inhibitor	218.101 (− − −) 5137.96	IC50 value of 105.6 µM in TE2 (squamous cell carcinoma) lines (Isozaki et al., 2014).	−1.255
Hexylcaine	NaChB	261.365 + 20.28	No clear evidence to use in the any cell line for cytotoxicity, however, one patent and a journal article claim that it may have anticancer activity (Gleich, 2002, Li et al., 2020).	0.086
5-hydroxytryptophan	Amino acid for the treatment of depression	220.228 (+ −) 3.42	Promotes immunity against cancer as indicated by a study, where an anti-tumor effect was observed with mouse xenograft model of colon-adenocarcinoma (Huang et al., 2022).	−0.767
Hydroxyzine	HRB	374.91 + 3.03	Cell death reported when tested on breast cancer cell lines (Shakya et al., 2022).	0.146
Hyoscyamine	AchRB	289.375 + 436.80	One study predicted possible anticancer effect from clinical data in non-Hodgkin lymphoma (Friedman et al., 2009).	0.234
Ketotifen	HRA, phosphodiesterase inhibitor	309.43 + 0.43	Suppression of migration and invasion of MDA-MB-231 breast cancer and HT-1080 fibrosarcoma cancer cells (Kim et al., 2014); inhibition of prostate cancer cells in vitro and in vivo (Ji et al., 2023).	0.283
Ketorolac	COX-I	255.273 (−) 255.27	Apoptosis induction in RCC (Sonawane et al., 2023); in vivo reduction of ovarian tumor burden (Grimes et al., 2021); reduce angiogenesis in TNBC xenograft (Liu et al., 2020b); apoptosis in osteosarcoma cells (Zuckerman et al., 2019); reduced MG63 osteosarcoma cell viability (Luna-Bertos et al., 2013).	−0.022
L-arginine	Amino acid	174.20 (+ + −) 6742.41	Inhibition of proliferation of colorectal cancer, clinical data (Hu et al., 2001).	−1.089
L-glutamine	Amino acid	146.146 (+ −) 310.98	In vivo suppression of rat mammary adenocarcinoma in Walker-256 xenograft (Martins et al., 2016).	−0.653
L-methionine	Amino acid	149.21 (+ −) 44.68	Inhibition of growth of human pancreatic cells (Benavides et al., 2014).	−0.35
Lornoxicam	COX-I	371.81 (−) 12.91	An in-silico study predicts its efficacy in colorectal cancer (Unal and Gov, 2023).	−0.966
Loxoprofen	COX-I	246.306 (−) 246.31	Decreasing tumor growth in lung carcinoma LLC and KLN205 models in mouse (Kanda et al., 2003).	0.072
Metergoline	DRA, SRB	403.526 + 0.54	Inhibition of 5HT-7 receptor in TNBC, this receptor has possible influence on cancer proliferation (Cınar et al., 2022); with another similar study on small cell lung carcinoma lines (Cattaneo et al., 1994).	0.39
Metixene	AchRB	309.47 + 3.38	Induction of apoptosis in vivo in a breast cancer mouse model (in mouse brain) (Fares et al., 2023).	0.923
Nalbuphine	Opioid receptor modulator	357.45 + 22.96	Suppression of breast cancer stem cells (Yu et al., 2019) and leukemia stem cells (Xiao et al., 2023).	−0.514
Naproxen	COX-I	230.263 (−) 119.14	Cytotoxicity in colorectal adenocarcinoma, hepatocellular carcinoma, mammary gland carcinoma, epithelioid cervix carcinoma, lung carcinoma, and epidermoid larynx carcinoma cell lines (Motawi et al., 2014).	0.348
Nedocromil	HRB	371.345 (− −) 371.35	One patent claiming that it improves pancreatic cancer (Owen, 2022).	−0.97
Nefopam	COX-I	253.345 + 1.15	Suppression of murine aggressive fibromatosis tumor (Poon et al., 2012).	0.348
Nortriptyline	TCA	263.384 + 21.63	Clinical trial in small cell carcinoma, inhibitory effect on pineoblastoma and melanoma cell lines (Petrosyan et al., 2022).	0.823
Opipramol	Sigma receptor antagonist	363.505 + 0.21	Antitumor efficacy in melanoma cell lines (Nordenberg et al., 2005).	0.652
Ozagrel	Thromboxane synthase inhibitor	228.251 (−) 11.86	Effect on lung cell carcinoma, cell viability assay: 48 h IC50 < 1 mM (Liu et al., 2016).	0.083
Phenelzine	MAO-I	136.198 + 69.51	Inhibition of growth on prostate cancer cells (Gaur et al., 2019).	−0.103
Piperacetazine	DRB	410.58 + 1.60	Inhibition of the oncogenic fusion protein PAX3-FOXO1 in alveolar rhabdomyosarcoma (Nakazawa et al., 2023).	0.008
Piperidolate	AchRB	323.436 + 1.50	A study suggested it as a candidate for colonic adenocarcinoma through small molecule connectivity mapping (Wang et al., 2023a).	0.476
Piribedil	DRA	298.346 + 1.46	At a dose of 100 mg/kg, inhibition of tumor growth in mouse xenografts with MLL-r leukemia cells (Zhang et al., 2018).	−0.026
Pizotifen	SRB	295.44 + 0.39	Significant decrease of proliferation of HCT116 colorectal cancer cells at 15, 20 and 25 μM (Piao and Shang, 2019).	0.732
Promazine	DRB	284.42 + 1.34	Cytotoxic to KHT fibrosarcoma cell lines (Lehnert, 1986).	0.853
Promethazine	HRB	284.42 + 0.87	Apoptosis in cells of chronic myeloid leukemia, colorectal cancer, small cell lung cancer and pancreatic ductal adenocarcinoma (Medeiros et al., 2020, Mehrabi et al., 2023). No effect on growth of glioma U87 cells with concentration of 100 nM, 1 μM, and 10 μM (Poteet et al., 2013).	1.131
Ropinirole	DRA	260.381 + 260.38	IC50 43.10 ± 9.58 μg/ml for breast cancer cell lines (Rymbai et al., 2022).	0.148
Serotonin	Neurotransmitter	176.219 + 462.62	Two publications claimed anti-tumor efficacy in rat model of M−1 (leukemia), PC-1 hepatoma, and 45 (hybridoma) at 10 mg/kg (Mal'tseva, 1968, Vinnitskii, 1970).	−0.263
Sulpiride	DRB	341.43 + 126.11	An in silico study predicts it as a candidate for colorectal cancer (Unal and Gov, 2023).	−1.062
Taurine	Antioxidant	125.14 (+ −) 1241.32	Reports of activity in cervical, lung and colorectal cancer (Ma et al., 2022).	−0.446
Terguride	DRA SRB	340.471 + 0.72	Induction of apoptosis and suppression of tumor growth in a prolactinoma model in rat pituitary gland (YONEZAWA et al., 1997).	0.086
Tilorone	Interferon inducer	410.56 (+ +) 4.68	One Phase II trial with breast carcinoma (Kuperminc and Gelber, 1976), antitumor effects in several rat studies (Basley et al., 1981), Lewis lung carcinoma and B-16 melanoma (Morahan et al., 1974).	0.057
Tizanidine	Adrenergic agonist	253.71 + 1.09	When loaded in nanoparticles, cytotoxic to MCF-7 breast cancer, HOP92 lung cancer, and A549 lung cancer cell lines (Sinha et al., 2022); inhibition of proliferation of small cell lung cancer cells A549 (Zhao et al., 2020), cytotoxic to osteosarcoma cell lines (Xing et al., 2019).	0.084
Tolperisone	Voltage-gated NaChB	245.366 + 4.16	Inhibition of A375 (melanoma), 8505C (thyroid neoplasm), AGS (gastric adenocarcinoma), and RKO colon cancer cell lines (Jiang et al., 2023).	0.457
Trazodone	SRB, SRI, Adrenergic antagonist	371.87 + 0.97	Gene expression profiling and use of drug expression profiles have yielded it as a candidate for atypical meningioma (Zador et al., 2018).	0.497
Venlafaxine	SNRI	277.408 + 30.37	Effects on melanoma, including apoptosis in MV3 human melanoma cell (Niu et al., 2023).	0.254
Xylazine	Adrenergic receptor agonist	220.33 + 8.23	Overexpression of ADRA2A inhibited growth of ovarian cancer cell lines and xylazine was found to be an ADRA2A agonist (Albanna et al., 2023).	0.421
Zaltoprofen	COX-I	298.36 (−) 43.20	Growth inhibition in chondrosarcoma (Higuchi et al., 2023). Evidence of chondrosarcoma patient survival (Higuchi et al., 2018).	−0.138

MW, molecular weight; a, charge at pH 7.4; b, solubility at pH 7.4 (mg/ml); DRA, Dopamine receptor agonist; DRB = Dopamine receptor antagonist; SRA, serotonin receptor agonist; SRB, serotonin receptor antagonist; NRI, norepinephrine reuptake inhibitor; SNRI, Serotonin and NE reuptake inhibitor; NE, norepinephrine; GluRB, Glutamate receptor antagonist; AchRA, acetylcholine receptor agonist; AchRB, acetylcholine receptor antagonist; NaChB, Sodium channel blocker; CaChA, calcium channel activator/agonist; CaChB, calcium channel blocker; SSRI, selective serotonin reuptake inhibitor; HRA, histamine receptor agonist; HRB, histamine receptor antagonist, TCA, tricyclic antidepressant; LA, local anesthetics; BBB, blood brain barrier; COX-I, cyclooxygenase inhibitor; MAO-I, monoamine oxidase inhibitor; GBM, Glioblastoma, 5-FU, 5 fluorouracil, TNBC, Triple negative breast cancer, ER, estrogen receptor; HCC, hepatocellular carcinoma; NB, neuroblastoma; IC50, half-maximal inhibitory concentration.

### 2.5 Search timeline

The timeline of search was from January 10 to April 30, 2024. It is then updated from October 7 to October 14, 2024.

## 3 Results

### 3.1 Screening for chemically suitable drugs

**Fig. 2:**
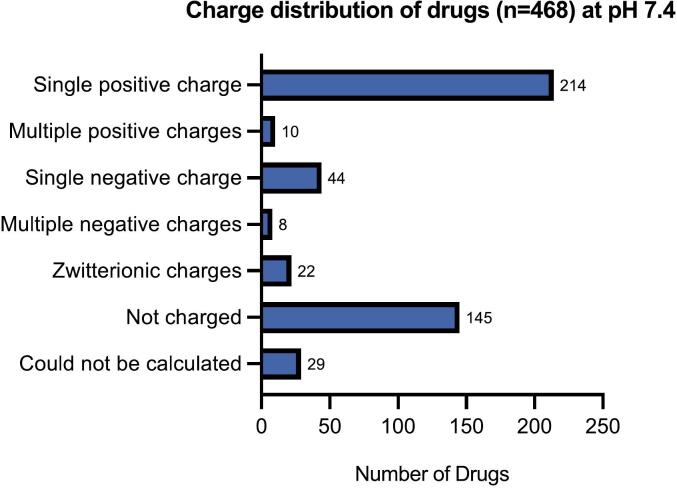
Number of drug compounds having different charges at pH 7.4 (calculated using Chemicalize software).

The 468 drugs from the 'neurology/psychiatry' category were first analyzed for their charge at pH 7.4 (Fig. 2). The number of compounds displaying charges were as follows: single positive (n = 214), multiple positives (n = 10), single negative (n = 44), multiple negatives (n = 8) and zwitterionic (n = 22). In total 283 compounds were charged (few multiple positives, multiple negatives and zwitterionic were not mutually exclusive; and duplicates (n = 11) were removed), while less than half of the compounds were not charged at pH 7.4 (n = 145), and a few (n = 29) could not be calculated by Chemicalize (Table S2).

### 3.2 Evidence of anticancer activity of neuroactive drugs

**Fig. 3:**
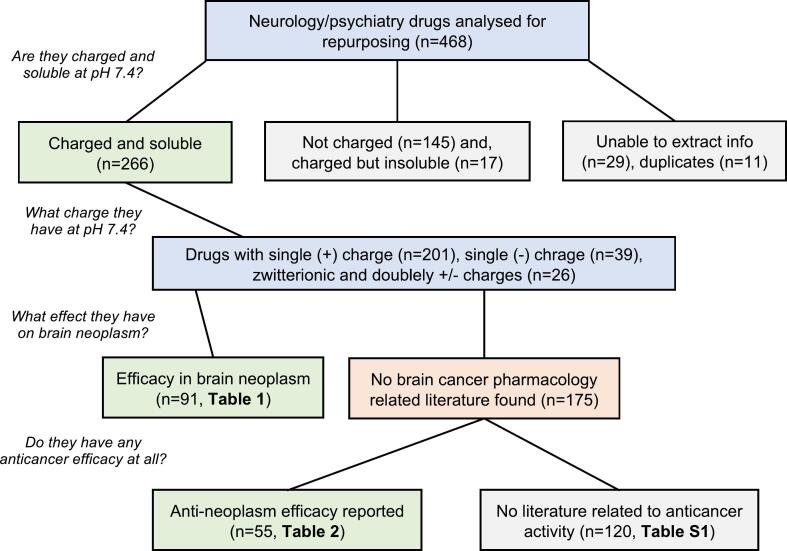
Schematic diagram for screening of neuroactive drugs for anti-brain cancer and overall anticancer activity.

283 of the 468 drugs were found to be charged at physiological pH. However, 17 of them were poorly soluble in aqueous media (<0.01 mg/ml) at pH 7.4 (Table S3), and 266 of the charged drugs were selected for further screening for their anticancer activity. From the extensive database search as indicated in the method section and in the Fig. 1, it was found that brain tumor inhibitory action was reported for 91 agents (Table 1). From the other 175, another 55 compounds were found to have reported anticancer effects for other forms of neoplasms (Table 2). Fig. 3 illustrates the numbers of drugs fitting the various categories during the process of screening.

**Fig. 4:**
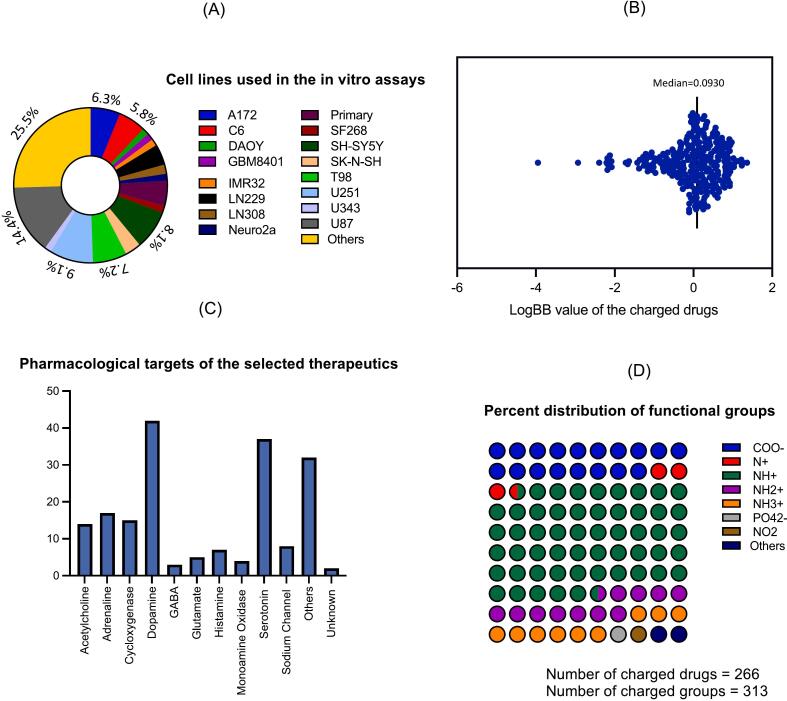
Different characteristics of the drug molecules and their evidence of anticancer efficacy: The distribution of in vitro cell models used in the studies found in the literature (n = 91) (A), logBB distribution of charged drugs of the 'neurology/psychiatry' category (n = 283) (B), molecular target distribution of the charged and soluble repurposing therapeutics (n = 266) (C), and distribution of functional groups responsible for charge in the molecules (n = 266) (D).

The evidence of brain cancer activity of the screened compounds from the systematic literature search are summarized in Table 1 and, the evidence of the overall anticancer activity is listed in Table 2. The main pre-clinical evidence of these drugs being repurposed as a brain cancer therapeutics were from in vitro cell culture studies and the most prevalent cell lines used were glioma cell lines U87, T98, U373, A172, and C6, and the neuroblastoma cell line SH-SY5Y (Fig. 4**A**). Use of primary brain cancer cells were found only in 5.3 % of the in vitro assays considered in this review (Fig. 4**A**).

The therapeutic class by receptor activity of individual drugs are noted in the second column of Table 1, and Table 2, while molecular weight, charge and solubility at physiological pH, evidence of anticancer efficacy and BBB permeability profile were noted in the subsequent columns. 168 of the charged drugs from the 'neurology/psychiatry' category was found to have a positive logBB value indicating their likelihood of crossing the BBB, while 115 of them having negative values (Fig. 4**B**).

Among the therapeutic categories of charged drug candidates with potential for repurposing, antipsychotics (n = 29), drugs for neurodegenerative disorders (n = 25), antidepressants (n = 23) and anti-inflammatory drugs (used in the treatment of symptoms of neurology diseases, n = 17) were prominent (Table S4). Target-wise, drugs acting on dopaminergic (n = 42) and serotonin (n = 37) systems were the most frequent showing anti-tumor activity, followed by agonists/antagonists of adrenergic (n = 17) and cholinergic systems (n = 14) and cyclooxygenase inhibitors (n = 15) **(**Fig. 4**C)**. Among the other therapeutic class, anesthetics, anti-seizures, antihistamines used for motion sickness, anti-stroke and smoke cessation agents were mentionable.

Most of the identified drugs carry positive charges in aqueous media at pH 7.4 (n = 246), and the bulk of the positive charges are due to primary, secondary, tertiary or quaternary amines, comprising 78.28 % of all charged compounds (Fig. 4**D**). The carboxylate ion (COO–), on the other hand, was the predominant negatively charged functional groups (17.9 %) (Fig. 4**D**).

#### 3.2.1 Mechanism of anti-brain tumor efficacy of the screened drugs

Many of the candidates from the list act on the dopaminergic system (Fig. 4**C**). For example, many of the antiparkinsonian drugs listed, such as L-dopa, apomorphine, carbidopa, monoamine oxidase inhibitors (MAO-I) and catechol-O-methyl transferase inhibitors (COMT-I), were originally designed to mimic dopamine or increase dopamine levels in the brain. However, the potential correlation between dopamine and antitumor efficacy of those drugs has been investigated. In fact, one study postulated that because of their dopaminergic therapy, Parkinson's patients have a reduced incidence of cancer (Lalonde and Myslobodsky, 2003), and in addition, dopamine agonists have shown antiproliferative effects across different in vitro and in vivo models (Rubí and Maechler, 2010).

However, Clement et al. argued, while dopamine itself can be cytotoxic to neuronal cells in vitro at certain doses, this effect might stem from some reactive intermediates like dopamine-o-quinone in the culture medium rather than the direct dopamine receptor activation (Clement et al., 2002). To add to this point, a study demonstrated that, dopamine receptor D2 (DRD2) silencing in U87 glioma cells significantly reduces cell viability, implicating necessity for dopamine signaling in glioma survival (Li et al., 2014).

Dopamine-mimicking compounds such as apomorphine can act as an anticancer agent independently of dopamine signaling, targeting metabolic pathways (Lee et al., 2016b). Similarly, carbidopa and benserazide, structurally related to dopamine, may exert antitumor effects by acting on different proteins like aryl hydrocarbon receptor, hexokinase 2 and cystathione B-synthase (Ogura et al., 2017). MAO-Is, such as tranylcypromine and phenelzine are thought to have anticancer effects in GBM by influencing an epigenetic modifier named lysine-specific demethylase-1 (Abadi et al., 2022). These examples highlight the diverse mechanisms through which dopamine-related drugs work as cancer therapeutics.

However, the anti-dopaminergic effects of antipsychotics have been studied and found to be associated with their anticancer efficacy. Multiple independent studies showed that dopaminergic receptors such as DRD2 and DRD3 are overexpressed in cancer cells including glioma cells and they correlate with the progression of the disease (Li et al., 2014, Sachlos et al., 2012, Williford et al., 2021). In 2017, a screening of 80 dopaminergic drugs on 3 different GBM cell lines concluded trifluoperazine, a DRD2 antagonist as the top candidate (Pinheiro et al., 2017). In addition, a review published in 2019 showed that at least 12 selective DRD2 antagonists showed efficacy against in vivo tumor models across the literature (Weissenrieder et al., 2019).

The anticancer effects of antipsychotics may also stem from their physicochemical properties. A 2024 study generated a 3D graph to analyze steric hindrance, pKa, and lipophilicity, concluding that weakly basic (pKa 8–10), highly lipophilic (logP 4–6) drugs, and drugs with low steric hindrance (k value 2–4) are correlated with anticancer efficacy (Jacob et al., 2024). With specific focus on the anticancer effect of a typical antipsychotic, perphenazine, the authors showed that the drug caused membrane damage in the lysosome, releasing enzymes in the cytoplasm and eventually killing the GBM cell (Jacob et al., 2024). In the current screening, 12 atypical antipsychotics and 16 typical antipsychotics were included. Interestingly, according to our use of Chemicalize, all typical antipsychotic included (except one) have a logP value higher than 3.6 (lipophilic) and most of them have pKa values over 8.5 (Table S5). However, neurology drugs in general are associated with relatively high pKa compared to other drugs, and one study showed the pKa of most of the CNS drugs developed are within the range 7.5–8.5 (Manallack, 2007).

In case of antidepressant drugs, it is difficult to decide from the published literature, which pharmacological class would be more suitable for anticancer therapy. For example, in 2022, a study with 40 cell lines showed protriptyline, a tricyclic antidepressant (TCA) had better glioma sensitivity score (in comparison to TMZ) than 12 other psychoactive compounds (Lin et al., 2022). In addition, in a case-control study in Denmark, with a large sample (75340 controls and 3767 glioma patients), long term use of TCA was found to be inversely corelated with glioma, while in the same study, selective serotonin uptake inhibitors (SSRIs) were not found to have any correlation with glioma (Pottegård et al., 2016). In contrast, a screen with at least 67 neuroactive drugs, reported serotonin reuptake inhibitor vortioxetine as a top candidate, while other two top candidates, paroxetine and fluoxetine, were SSRIs (Lee et al., 2024).

There are many proposed mechanisms involving anticancer effects of antidepressants. Studies showed that imipramine, vortioxetine, sertraline and fluoxetine can inhibit autophagy in cancer cells by blocking mTOR, an autophagy regulator protein, hence induce autophagic cell death (He et al., 2023). Escitalopram, another SSRI, was reported to exert its anti-GBM efficacy by inducing autophagy in GBM801 and apoptosis in U87 and C6 cells in vitro (Chen et al., 2018, Dikmen et al., 2011). Many of the antidepressants, such as imipramine and amitriptyline, are reported to influence NF-xB gene expression in GBM cells and can partially reverse dysfunctional mitochondrial activity (Bielecka-Wajdman et al., 2018). Lee et al. proposed that the AP-1 and BTG upregulation is responsible for anticancer activity of vortioxetine (Lee et al., 2024). AP-1 transcription factors were thought to act as an oncogene, but recently, they are also found to suppress oncogenes, while BTG-1 has a proven growth suppressing role in tumors (Eferl and Wagner, 2003, Lee et al., 2024).

Anti-inflammatory drugs used to treat neuropathic pain, and other neurology symptoms, might have a direct correlation with their anticancer efficacy. It is apparent that prostaglandin E2 (PGE2) is directly responsible for tumorigeneses and anti-inflammatory drugs can bind to cyclooxygenase (COX)-2 to inhibit PGE2 signaling pathway (Cha and DuBois, 2007). Therefore, selective COX-2 inhibitors, such as parecoxib and celecoxib can reduce cell proliferation and migration of GBM cells (Li et al., 2017a, Sareddy et al., 2013). However, it is worth noting that, tolfenamic acid, a selective COX-1 inhibitor was also reported to have an apoptotic pathway for inhibiting the neuroblastoma and medulloblastoma cell lines in different studies (Eslin et al., 2013a, Eslin et al., 2013b).

O6-methylguanine-DNA methyltransferase (MGMT) is a DNA repair enzyme that removes the alkyl groups at the O6‐guanine position induced by alkylating drugs such as TMZ, thereby inducing drug resistance (Richard et al., 2020). Some repurposed therapeutics, such as anti-epileptic drug valproic acid can sensitize the tumor cells to the standard chemotherapeutic TMZ, by downregulating MGMT expression (Ryu et al., 2012). Valproic acid and other antiepileptic drugs, such as tiagabine, are reported to inhibit T98 and U87 as independent therapeutics as well (Lee et al., 2016a).

#### 3.2.2 Ranking the drug molecules based on efficacy

It is difficult to formulate a scale to rank the drugs from the results of the preclinical studies as different drugs can produce different efficacy results in different cell lines. For example, in a MTT viability study, trifluoperazine showed a lower IC50 (4.2 μM) than 12 other compounds, including thioridazine (8.2 μM) in the melanoma cell line B16 (Xia et al., 2021). However, in another MTT viability assay in the GBM411 cell line, thioridazine showed higher efficacy at a 10 μM dose than trifluoperazine (Cheng et al., 2015). Furthermore, perphenazine showed better viability profile across 3 GBM cell lines than thioridazine in another study (Jacob et al., 2024).

However, for the therapeutics having potential for repurposing in brain cancer, it is at least possible to tabulate them in terms of the clinical progress they made (Table S6). The idea was that top candidates should either have a good clinical outcome, or otherwise compelling in vivo evidence to show prolonged survival and reduction of the tumor volume. Below is the discussion of a few of those top candidates’ story which underwent a clinical trial and their rationale for their use in brain cancer (clinical progress of are summarized in Table S6).

*Valproic acid*: Anti-seizure medications, such as valproic acid, is commonly prescribed with brain tumors to treat or prevent epileptic symptoms of the patients (Aronica et al., 2023). Inhibition of histone acetylation via histone deacetylase and upregulation of cell cycle inhibitor protein p21 is thought to be one of the reasons of its anticancer activity (Chang et al., 2017). In a study, authors found the IC50 of the drug in U87MG GBM cells is 808.82 μg/ml and T98 is 652.78 μg/ml, which is quite high for an anticancer drug (Lee et al., 2016a). However, in different studies in a clinical setting, patients receiving valproic acid for treating seizure symptoms showed prolonged overall survival (Rudà et al., 2016). In fact, in a phase II study completed in 2014, patients receiving the drug showed overall median survival around 29.6-month, while administered with TMZ and concurrent radiotherapy (Krauze et al., 2015). Another similar phase III study was supposed to finish in 2023, yet no result is posted online (NCT03243461).

*Atorvastatin*: The Broad Institute Drug Repurposing Hub included atorvastatin in the neurology category because of its indication in stroke. Statins, like anti-seizure medications, also inhibit histone acetylation and can increase p21 levels (Chang et al., 2017). In an in vitro study in a spheroid mouse glioma CT-2A model, 32 μM atorvastatin alone could inhibit more than 95 % of the cells after 72 h (Luebtow et al., 2020). In an in vivo brain tumor model with C6 glioma cells, atorvastatin reduced the tumor volume significantly (Goodarzi et al., 2020). A phase II study with 36 patients reported 19.9 months of survival, but progression-free survival was 7.6 months, which barred the study to reach its primary endpoint (Altwairgi et al., 2019). Another phase II interventional study is ongoing in China (recruiting) (NCT06327451).

*Sertraline*: Sertraline is one of the antidepressants generally used in brain cancer therapeutics to deal with the associated anxiety (Gramatzki et al., 2020). Preliminary studies indicated that antidepressant sertraline can induce autophagic cell death in cancer cells by blocking Akt/mTOR pathway to induced autophagy-mediated cell death (He et al., 2023). It was one of the drugs in the 9 repurposing adjuvant drugs in the CUSP9v3 regimen, which was tested in a phase Ib/IIa trial. The trial did not reach its endpoint but showed some positive signs such as 12 months of progression free survival in 50 % of the patients (Halatsch et al., 2021).

*Chlorpromazine*: In in vitro cell studies, chlorpromazine, a drug used in schizophrenia treatment, showed efficacy against multiple GBM cell lines, such as T98G, U-251 MG and U-87 MG, in different independent studies (Matteoni et al., 2021a, Matteoni et al., 2021b, Shin et al., 2013). This drug also reduced the tumor volume in a nude mouse model with U-87 xenograft (Shin et al., 2013). In 2023, the results of a phase II clinical trial with chlorpromazine as an adjuvant chemotherapy to TMZ was published. The trial was designed for MGMT unmethylated patients. The overall survival was found to be 15 months (Pace et al., 2023).

*Imipramine*: Imipramine, a tricyclic antidepressant, is used in GBM and other brain cancers to treat cancer-associated psychiatric comorbidity (Gramatzki et al., 2020). The number of articles that have investigated pre-clinical anti-brain cancer efficacy of imipramine is limited. However, there is one study that compared rat astrocytes with C6 and U87 cell lines in terms of cell death analysis and clonogenic survival assay. In 40 and 60 μM doses, imipramine affected C6 and U87 cells, but not astrocytes (Jeon et al., 2011). There is an ongoing phase 2 clinical trial in USA (to be finished in 2026), where in the study arms, imipramine is used as an adjuvant to lomustine (NCT04863950). However, within the search range of this paper, there was no published evidence of these two drugs in combination showing better efficacy except for one conference abstract published in 2023 (Venkata et al., 2023).

*Chlorogenic Acid*: This antioxidant was included in the 'neurology/psychiatry' category because of its indication in headache. A study showed that it can initiate cell differentiation in neuroblastoma cells Be(2)-M17 and SH-SY5Y, so that the cells start behaving like neurons (You et al., 2023). In the in vivo studies, it showed reduced tumor volume in human GBM cell U372 in 10 mg/kg and 20 mg/kg (Xue et al., 2017). One phase I clinical trial used chlorogenic acid as a monotherapy in patients with different grades of glioma, achieving a median survival of only 11.3 months for all patients and 9.5 months for high grade glioma (Kang et al., 2023). China’s regulatory authority approved a phase II clinical trial based on that data (Li et al., 2024b).

*Memantine*: Memantine is a glutamate inhibitor used in the treatment of Alzheimer’s disease. In the preclinical studies, memantine alone showed reduced viability in T98G and U87 GBM cell lines, with an IC50 of 0.5 mM for both cultures (Albayrak et al., 2021). A phase Ib/II randomized trial alongside chemotherapy and radiotherapy for newly diagnosed GBM is currently ongoing (Mastall et al., 2024). One of the other charged repurposed drugs, gabapentin, is also included in that trial (Mastall et al., 2024).

In addition to therapeutics mentioned above, aspirin and propranolol (whose antitumor effects are detailed in Table 1), along with five other medications used as adjuvant with TMZ, were explored in a phase I clinical trial. Although the trial results hinted at increased survival with the treatment regimen, the findings were not statistically significant (O'Rawe et al., 2022). The dopamine D2 receptor antagonist haloperidol is currently in a phase II clinical trial (in combination with TMZ), which is estimated to be completed in 2028 (NCT06218524). Finally, a phase I clinical trial aimed at determining the safety of the antidepressant maprotiline in combination with tamoxifen and TMZ was initiated but withdrawn before enrolling any patients, for unknown reasons (NCT04200066).

### 3.3 Evidence of affinity-based loading and controlled release of the selected therapeutics

Electrostatic interactions can be the governing factor for loading and slow release of the biomolecules from several charged polymeric carriers (Hakami et al., 2024, Pakulska et al., 2016). In the case of small molecules, there seems less evidence of sustained drug release based on electrostatic affinity-based interactions between the drug and the delivery system. However, Khachani et al analyzed the release of doxorubicin (cationic), acridine orange (cationic) and Alexa 546 (anionic) from either negatively charged nanosilicate clay hydrogels or non-charged polyethylene glycol-based polymers. Their work showed slow release, only for combinations where the molecule and the delivery system were of opposite charges (Khachani et al., 2022). Furthermore, the electrostatic affinity of cationic doxorubicin to negatively charged heparin has been modelled in silico. Subsequent in vitro analysis showed, 42-day doxorubicin release from the anionic cryogels (Newland et al., 2020). In another study, an anesthetic bupivacaine was released for 60 days from a microgel-hydrogel composite, and the loading and release was based on electrostatic affinity (Sivakumaran et al., 2011).

The drugs included in this review contain positive, negative or sometimes both charges (Table 1, Table 2) and evidence from the literature described below shows that some candidates can achieve a controlled release by affinity-based interactions with a carrier.

In one study, a positively charged candidate amantadine, an antidyskinetic drug used in the treatment of Parkinson's disease, was formulated to form an inclusion complex with a magnetic carboxymethylated β-cyclodextrin carrier. The authors attributed the high loading capacity of the carrier to the electrostatic attraction of the cationic drug to negative –OH and –COOH groups of the carrier (Hadadian et al., 2025). Clozapine, a cationic drug mainly used in schizophrenia, was incorporated into a Pluronic gel system for sustained release of the drug. Although there are no direct charges expressed in the gel system, the authors reported dipole–dipole interactions with clozapine and Tween 80 used in the formulation, which might facilitate the sustained release of the formulation (Abdulla et al., 2021). Another study showed controlled release of cationic citalopram and trazodone (antidepressants) from cross-linked hydrogels with l-phenylalanine or l-valine residues, due to the COO– on the amino acid residue (Casolaro and Casolaro, 2015). In addition, drug loading and release of positively charged amitriptyline (TCA), chlorpromazine (typical antidepressant) and doxepin (TCA) were reported to be affected by their electrostatic interactions with negatively charged polyacrylate microgels, hyaluronic acid microgels and negatively charged DCbead^TM^ in a drug-eluting bead system (Al-Tikriti and Hansson, 2020, Wanselius et al., 2023).

For anionic drugs, Dragan et al. showed controlled release of diclofenac (an anti-inflammatory drug) based on an electrostatic attraction, especially at a lower pH, and a release time of at least 400 min at pH 7.4. The carrier system used in this study was a polymeric hydrogel system with a cationic polymer named poly(N,N-dimethylaminoethyl methacrylate) (Dragan and Cocarta, 2016). In a 2022 study, authors loaded naproxen, an anionic anti-inflammatory drug in 6 types of mesoporous silica particles, where (3-aminopropyl)triethoxysilane was the only positively charged carrier with a –NH_3_ + group. Interestingly, the drug release from (3-aminopropyl)triethoxysilane was lower after 48 h compared to other carriers, presumably due to the retention of the anionic drug by the electrostatic affinity of the amine group (Zauska et al., 2022).

## 4 Challenges and limitations

Each step of this systematic screening had different challenges and limitations. Starting from the repurposing database: 'neurology/psychiatry' category of Board Institute Repurposing Hub contains not only drugs that are used in anxiety/psychosis/dementia but also drugs that are prescribed for a nervous system condition, such as analgesics and anti-inflammatory drugs suggested in neuropathic pain, and headache. Even, some molecules which are used in treating toxicity associated with drugs acting on nervous system, like chelating agent EDTA, were in the database. However, we didn’t exclude any compounds in the beginning of the search as the end target was to find a suitable charged candidate for brain cancer.

Nevertheless, in the end of the screening, we had to exclude some possible candidates with the potential to be repurposed, due to them not having charge. To give an example, disulfiram, a drug used in chronic alcoholism was in the repurposing database initially but was excluded as it is not charged at pH 7.4 (Table S2). However, a nano-emulsion formulation of the drug improved survival in a pre-clinical glioblastoma model following nose to brain delivery, showing its potential to be repurposed for brain cancers (Qu et al., 2021).

From the literature most of the candidates that showed anticancer efficacy were from in vitro studies. Not only do those studies differ from one cell type to another, but also their anticancer effect may vary depending on the passage number of the cells (Chang-Liu and Woloschak, 1997). Contrasting evidence also exists; for example, citalopram, imipramine and desipramine, all showed antitumor efficacy in vitro (different sources mentioned in the Table 1), but another study reported that GDNF levels are increased in C6 glioma cells by all the three candidates, which might influence the growth of the tumor (Golan et al., 2011). In addition, it is difficult to predict the response of the drug due to the variation of the tumor genotypes in patients. Even the TMZ, the standard therapeutic for GBM, is more effective in specific genotypic group of patients who contains methylated-MGMT promoter gene than in patients with non-methylated MGMT promoter (Fernandes et al., 2017). Drug specific factors may as well contribute to the observed gap between experimental promise and therapeutic performance. Disulfiram is a notable example of a drug that, despite encouraging preclinical findings, has failed to demonstrate consistent clinical efficacy—largely due to its pharmacokinetic drawbacks (Benko et al., 2023). Therefore, in selecting the better candidates, clinical studies and in vivo studies were given the most priority.

Another potential limitation of repurposing current therapeutics is that the dose required for an anticancer effect may be many times higher than that used in their current indication, potentially compromising their established safety profile. Repurposed anti-malarial drugs chloroquine and hydroxychloroquine, for example, can be associated with side effects in regular doses and can be lethal in certain doses (>50 mg/kg) (Abdel-Aziz et al., 2022). Local delivery to the brain tumor site might offer an alternative way to achieve a high enough concentration of the repurposed drugs at the site of action to be tumor-inhibitory while avoiding the system-wide dose-related side-effects (Bastiancich et al., 2021a).

While local delivery of chemotherapeutics in preclinical studies has showed a favorable therapeutic outcome (Bastiancich et al., 2021a, Wang et al., 2023b), long-term release of the therapeutics above the effective dose is necessary to get the optimal outcome. For macromolecules like proteins, long term affinity-based release from polymeric systems up to 28 weeks was observed before, while for small molecules obtaining a similar result might be a challenge (Hakami et al., 2024). Therefore, in future, there is a need for experimental evidence for long-term local release of repurposed therapeutics for the treatment of brain cancer. This study provides a foundation for selecting suitable neurology drug candidates for such efforts.

## 5 Conclusion

This review has attempted to find charged small molecules suitable for repurposing for brain cancer therapy. The idea behind the search was, if a drug can be electrostatically paired with a charged drug delivery system, the partnership can achieve an affinity-based controlled release in physiological pH and thus, can be directed for local delivery in the glioblastoma resection cavity after surgery. To search for a suitable repurposed candidate from neuroactive drugs, the Board Institute Repurposing Hub was consulted and their physicochemical (charge, solubility, lipophilicity), pharmacokinetic properties (BBB penetration and distribution) and pharmacodynamic (anticancer efficacy) were compiled. A total of 91 positively, negatively, or zwitterionic charged molecules were identified from the literature as having anti-brain tumor efficacy, predominantly in pre-clinical settings. While some of the candidates were found to be in clinical trial, most of the results are not complete yet. This review may serve as a resource for drug delivery scientists exploring affinity-based drug delivery of repurposed anticancer agents.

## CRediT authorship contribution statement

**Sabarni Sarker:** Writing – original draft, Resources, Methodology, Investigation, Formal analysis, Data curation. **Ben Newland:** Writing – review & editing, Validation, Supervision, Methodology, Funding acquisition, Conceptualization.

## Declaration of competing interest

The authors declare that they have no known competing financial interests or personal relationships that could have appeared to influence the work reported in this paper.

## Data Availability

No data was used for the research described in the article.
