# Supplementary material for: Repurposing neurological drugs for brain cancer therapeutics: A systematic approach to identify charged molecules for affinity-based local drug delivery systems
Source: Int J Pharm. 2025 Sep 15;682:125935. doi: 10.1016/j.ijpharm.2025.125935 (PMC13197229; doi:10.1016/j.ijpharm.2025.125935)
Supplement: Supplementary Data 1 [file mmc1.docx]

**SUPPLEMENTARY INFORMATION**

**Repurposing neurological drugs for brain cancer therapeutics: Systematic identification of charged molecules for affinity-based drug delivery systems**

Sabarni Sarker^a,b^, Ben Newland^a,c^ *

a School of Pharmacy and Pharmaceutical Sciences, Cardiff University, King Edward VII Avenue, Cardiff, CF10 3NB, United Kingdom

b Department of Pharmacy, Jagannath University, Dhaka 1100, Bangladesh

c Leibniz-Institut für Polymerforschung Dresden e.V., 01069 Dresden, Germany

** Corresponding author*

**Table S1**. List of charged 'neurology/psychiatry' drugs from with no evidence of anticancer activity from the literature by the defined search methodology.

| Drug | Class | MW | Charge^a^ | Solubility^b^ | LogBB |
| --- | --- | --- | --- | --- | --- |
| Acamprosate | GluRB | 181.21 | (-) | 44445.19 | -0.536 |
| Acepromazine | DRB | 326.46 | + | 0.62 | 0.482 |
| Acetophenazine | DRB | 411.56 | + | 3.94 | -0.080 |
| Acetyl-l-leucine | AA | 173.21 | (-) | 427.66 | -0.323 |
| Adiphenine | AchRB | 311.43 | + | 4.33 | 0.617 |
| Alimemazine | HRA/HRB | 298.45 | + | 0.83 | 0.866 |
| Almotriptan | SRA | 335.47 | + | 169.11 | -0.030 |
| Amfenac | COX-I | 255.27 | (-) | 255.27 | -0.150 |
| Amifampridine | K channel blocker | 109.13 | + | 12105.93 | -0.487 |
| Aminohydroxybutyric acid | GABA antagonist | 119.12 | (+ -) | 681.07 | -0.724 |
| Amisulpride | DRB | 369.48 | + | 47.11 | -1.145 |
| Amperozide | DRB | 401.50 | + | 0.13 | -0.129 |
| Arotinolol | adrenergic blocker | 371.53 | + | 2.99 | -1.099 |
| Articaine | LA | 284.37 | + | 9.56 | -0.207 |
| Atomoxetine | NE transporter inhibitor | 255.36 | + | 12.69 | 0.549 |
| Azaperone | DRB | 327.40 | + | 1.88 | 0.297 |
| Bendazac | COX-I | 282.30 | (-) | 275.35 | 0.435 |
| Benperidol | DRB | 381.45 | + | 0.07 | 0.177 |
| Bifemelane | Ach release enhancer | 269.39 | + | 269.39 | 0.720 |
| Bromperidol | DRB | 420.32 | + | 0.07 | -0.105 |
| Budipine | GluRB | 293.454 | + | 82.51 | 0.893 |
| Buspirone | SRA | 385.51 | + | 3.67 | -0.532 |
| Butacaine | LA | 306.45 | + | 30.16 | -0.120 |
| Caramiphen | Cholinergic antagonist | 289.42 | + | 4.12 | 0.545 |
| Cariprazine | DRB | 427.41 | + | 3.95 | 0.154 |
| Cinchocaine | LA | 343.47 | + | 0.39 | 0.367 |
| Clonixin lysate | COX-I | 262.69 | (-) | 14.48 | -1.638 |
| Clorotepine | adrenergic blocker, DRB | 344.90 | + | 0.18 | 0.805 |
| Clotiapine | Atypical antipsychotic | 343.87 | + | 0.01 | 0.322 |
| Cyamemazine | SRB | 323.46 | + | 0.51 | 0.414 |
| Cyclizine | HR modulator | 266.39 | + | 12.49 | 0.865 |
| Cyclobenzaprine | adrenergic agonist, SRA | 275.39 | + | 0.53 | 0.935 |
| Dalfampridine | K channel blocker | 94.12 | + | 3771.76 | -0.312 |
| Desvenlafaxine | SNRI | 263.38 | + | 59.09 | 0.048 |
| Dimenhydrinate | HRB | 469.97 | + | 0.19 | -1.361 |
| Dimethisoquin | LA | 272.39 | + | 0.30 | 0.751 |
| Diphenidol | AchRA | 309.45 | + | 14.82 | 0.575 |
| Dipyrocetyl | chelating agent | 238.19 | (-) | 238.20 | -0.614 |
| Dosulepin | TCA, SNRI | 295.44 | + | 0.44 | 0.921 |
| Doxylamine | HRB | 270.38 | + | 50.83 | 0.434 |
| Drofenine | antimuscarinic agent | 317.47 | + | 0.26 | 0.640 |
| Droxidopa | norepinephrine precursor | 213.19 | (+ -) | 72.86 | -1.202 |
| Dyclonine | sodium channel blocker | 289.42 | + | 0.79 | 0.633 |
| Edrophonium | SRA | 166.24 | + | 8.04 | 0.488 |
| Eletriptan | SRA | 382.52 | + | 8.04 | -0.030 |
| Eperisone | AchRB | 259.39 | + | 1.34 | 0.651 |
| Ephedrine | adrenergic agonist | 165.24 | + | 1113.17 | 0.135 |
| EDTA | chelating agent | 292.24 | (- - - -) | 99433.56 | -2.081 |
| Etilefrine | adrenergic agonist | 181.23 | + | 1765.39 | -0.17 |
| Felbinac | COX-I | 212.24 | (-) | 32.14 | 0.173 |
| Fenbufen | COX-I | 254.28 | (-) | 45.28 | 0.040 |
| Fentiazac | COX-I | 329.80 | (-) | 0.20 | 0.302 |
| Flibanserin | SRA | 390.40 | + | 0.14 | 0.360 |
| Flupirtine | GluRB | 304.32 | + | 0.16 | -1.164 |
| Fomocaine | NaCh modulator | 311.42 | + | 0.33 | 0.647 |
| Fosphenytoin | NaChB | 362.28 | (- -) | 362.28 | -1.238 |
| Frovatriptan | SRA | 243.31 | + | 243.31 | 0.065 |
| Galantamine | Ach. esterase inhibitor | 287.35 | + | 11.53 | 0.210 |
| Guanidinium | HSP inhibitor | 60.08 | + | 5530.79 | -0.155 |
| Hematoporphyrin | photosensitizer | 598.70 | (- -) | 598.70 | -2.124 |
| Iloperidone | DRB, SRB | 426.48 | + | 0.32 | -0.344 |
| Imidafenacin | AchRB | 319.40 | + | 2.18 | -0.366 |
| Isometheptene mucate^c^ | sympathomimetic | 141.26, 210.14 | 2 +,  (- -) | 492.65 | -2.183 |
| Itopride | DRB | 358.44 | + | 4.67 | 0.025 |
| Levodopa | Dopamine precursor | 197.19 | (+ -) | 52.94 | -0.848 |
| Levosulpiride | DRB | 341.42 | + | 126.11 | 1.027 |
| Lobeline | AchRB | 337.45 | + | 2.16 | -0.066 |
| Lofexidine | adrenergic agonist | 259.13 | + | 6.09 | 0.277 |
| Loxapine | TCA | 327.81 | + | 0.07 | 0.193 |
| Meclofenoxate | nootropic agent | 257.71 | + | 11.82 | 0.582 |
| Melperone | DRB, SRB | 263.36 | + | 9.86 | 0.447 |
| Meptazinol | opioid receptor agonist | 233.35 | + | 200.72 | 0.437 |
| Mesoridazine | DRB | 386.57 | + | 2.23 | 0.397 |
| Moprolol | adrenergic blocker | 239.31 | + | 251.26 | -0.069 |
| Naltrexone | opioid antagonist | 341.40 | + | 23.96 | -0.489 |
| Naratriptan | SRA | 335.46 | + | 178.09 | 0.031 |
| Nemonapride | DRB | 387.91 | + | 0.43 | 0.310 |
| Nicergoline | adrenergic blocker | 484.39 | + | 0.61 | 0.009 |
| Norepinephrine | adrenergic agonist | 169.18 | + | 3023.24 | -0.78 |
| Orphenadrine | AchRB | 269.38 | + | 3.58 | 0.793 |
| Oxybuprocaine | LA | 308.40 | + | 4.78 | -0.248 |
| Perospirone | DRB, SRB | 440.61 | + | 0.04 | 0.03 |
| Phenacaine | LA | 298.386 | + | 0.31 | 0.173 |
| Pimethixene | HRB | 293.43 | + | 0.16 | 0.787 |
| Pipamperone | DRB | 375.488 | + | 5.39 | -0.405 |
| Pipotiazine | DRB | 475.67 | + | 8.30 | -0.109 |
| Pirlindole | MAO-I | 226.323 | + | 6.07 | 0.536 |
| Pivagabine | corticotropin releasing factor receptor antagonist | 187.239 | (-) | 3191.94 | -0.264 |
| Pramipexole | DRA | 211.33 | + | 291.00 | 0.058 |
| Pramiracetam | AchRA | 269.389 | + | 1611.88 | -0.01 |
| Pranoprofen | COX-I | 255.273 | (-) | 255.27 | 0.007 |
| Pridinol | muscle relaxant | 295.426 | + | 16.41 | 0.566 |
| Procyclidine | AchRB | 287.447 | + | 6.45 | 0.575 |
| Profenamine | cholinergic antagonist | 312.48 | + | 0.75 | 0.836 |
| Proglumetacin | COX-I | 844.45 | + | 0.02 | -2.183 |
| Propoxycaine | LA | 294.395 | + | 14.40 | -0.274 |
| Proparacaine | LA | 294.395 | + | 14.41 | -0.206 |
| Reboxetine | adrenergic blocker | 313.397 | + | 0.13 | 0.21 |
| Rizatriptan | SRA | 269.352 | + | 163.57 | 0.233 |
| Rotigotine | DRA | 315.48 | + | 11.46 | 0.547 |
| Selegiline | MAO-I | 187.286 | + | 16.18 | 1.015 |
| Solriamfetol | dopamine reuptake inhibitor | 194.234 | + | 234.37 | -0.05 |
| Sumatriptan | SRA | 295.4 | + | 529.10 | -0.109 |
| Suxibuzone | COX-I | 438.48 | (-) | 108.61 | -1.017 |
| Tafamidis-meglumine | transthyretin amyloid inhibitor | 308.11 | (-) | 503.33 | -2.931 |
| Tandospirone | SRA | 383.496 | + | 2.03 | -0.499 |
| Thioproperazine | DRB | 446.63 | + | 5.33 | 0.083 |
| Thiothixene | DRB | 443.62 | + | 1.95 | 0.032 |
| Thonzylamine | HRB | 286.379 | + | 17.83 | 0.39 |
| Tianeptine | SSRE | (436.95) | (+ -) | 0.01 | -0.718 |
| Tiapride | DRB | 328.43 | + | 84.98 | -0.564 |
| Tinoridine | COX-I | 316.42 | + | 0.18 | 0.199 |
| Tolazoline | adrenergic blocker | 160.22 | + | 1145.18 | 0.129 |
| Triflusal | COX-I | 248.157 | (-) | 248.16 | -0.15 |
| Trihexyphenidyl | AchRB | 301.474 | + | 2.72 | 0.56 |
| Trimipramine | TCA | 294.442 | + | 1.59 | 0.92 |
| Tryptophan | SRA (partial) | 204.229 | (+ -) | 1.25 | -0.575 |
| Valbenazine | vesicular monoamine transporter inhibitor | 418.578 | (+ +) | 0.06 | 0.156 |
| Vedaprofen | COX-I | 282.383 | (-) | 0.06 | -0.016 |
| Vigabatrin | GABA aminotransferase inhibitor | 129.159 | (+ -) | 110.32 | -0.326 |
| Viloxazine | NE reuptake inhibitor | 237.299 | + | 7.86 | -0.034 |
| MW, molecular weight; a, charge at pH 7.4; b, solubility at pH 7.4 (mg/ml); DRA, Dopamine receptor agonist; DRB, Dopamine receptor antagonist; SRA, serotonin receptor agonist; SRB, serotonin receptor antagonist; NRI, norepinephrine reuptake inhibitor; SNRI, Serotonin and NE reuptake inhibitor; NE, norepinephrine; GluRB, Glutamate receptor antagonist; AchRA, acetylcholine receptor agonist; AchRB, acetylcholine receptor antagonist; NaChB, Sodium channel blocker; CaChA, calcium channel activator/agonist; CaChB, calcium channel blocker; SSRI, selective serotonin reuptake inhibitor; SSRE, selective serotonin reputake enhancer; HRB, histamine receptor antagonist, TCA, tricyclic antidepressant; LA, local anaesthetics; COX-I, cyclooxygenase inhibitor; MAO-I, monoamine oxidase inhibitor. | | | | | |

**Table S2**. List of non-charged and 'not calculated' drugs

| Not charged | Not calculated/not ionizable |
| --- | --- |
| adrafinil  afloqualone  afobazole  agomelatine  aminoglutethimide  ampiroxicam  apixaban  atipamezole  beclamide  bromantan  bromocriptine  butalbital  caffeine  capsaicin  carbamazepine  chlorobutanol  chlorphenesin  chlorphensin-carbamate  chlorzoxazone  clomethiazole  clopidogrel  dabigatran-etexilate  detomidine  dexamethasone  dexamethasone-acetate  dexmedetomidine  diclofenamide  difenpiramide  dimercaprol  doxapram  edaravone  edoxaban  epirizole  epomediol  eprobemide  ergotamine  eslicarbazepine-acetate  ethaverine  ethenzamide  ethosuximide  ethotoin  etifoxine  etizolam  etofenamate  etomidate  everolimus  felbamate  fenoverine  floctafenine  flopropione  flumazenil  halothane  hydrocortisone  ibudilast  ibuprofen-piconol  ibuproxam  idebenone  inositol-hexanicotinate  isocarboxazid  istradefylline  lamotrigine  levetiracetam  levomenol  levomenthol  lisuride  lofepramine  lomerizine  loratadine  mabuprofen  mannitol-D  medetomidine  melatonin  melevodopa  mephenesin  mephenytoin  mepivacaine  metaxalone  metharbital  methocarbamol  methsuximide  methyl-salicylate  methylergometrine  methylprednisolone  methylprednisolone-aceponate  methysergide  metocurine  mianserin  miconazole  mirtazapine  moclobemide  molindone  monomethyl-fumarate  morniflumate  moxaverine  nicotinyl-alcohol-tartrate  nifenazone  nonivamide  oxcarbazepine  oxethazaine  oxiracetam  paracetamol  phenacemide  phenazone  phenprobamate  phensuximide  phenylpiracetam  phenytoin  phloroglucin  physostigmine  piketoprofen  piracetam  prednicarbate  prednisone  primidone  propacetamol  propentofylline  propofol  proquazone  pyridoxal  pyritinol  quercetin  ramelteon  ramifenazone  riluzole  rivaroxaban  rotundine  rufinamide  salicylamide  scopolamine  setiptiline  sevoflurane  simvastatin  stiripentol  sulbutiamine  talniflumate  taltirelin  tasimelteon  teriflunomide  tetrabenazine  thiamylal  thiocolchicoside  thiopental  tofisopam  toloxatone  topiramate  trimetozine  valnoctamide  valpromide  vinburnine  vinpocetine  zonisamide | acetylcholine  atracurium  butylphthalide  cisatracurium  decamethonium  dimethyl-fumarate  diphemanil  disulfiram  enflurane  ephedrine-(racemic)  gallamine-triethiodide  isoflurane  levocarnitine-propionate  lithium-citrate  mebicar  megestrol-acetate  menadione  mestinon  methantheline  methoxyflurane  methylprednisolone-sodium-succinate  mivacurium  neostigmine  pancuronium  paramethadione  prednisolone-sodium-phosphate  succinylcholine-chloride  tiquizium  trimethadione |

**Table S3**. List of charged but water insoluble 'neurology/psychiatry' drugs.

| Drug | Class | MW | Charge^a^ | Solubility^b^ | LogBB |
| --- | --- | --- | --- | --- | --- |
| Alcuronium | Muscle relaxant | 666.90 | (+ +) | 0.0002 | -0.395 |
| Ambenonium | Cholinesterase inhibitor | 537.57 | + | 0 | 0.108 |
| Buclizine | Histamine antagonist | 433.04 | + | 0.001 | 1.264 |
| Dantrolene | Calcium channel blocker | 314.26 | (+ -) | 0.003 | -0.985 |
| Dihydroergocristine | Adrenergic antagonist | 611.74 | + | 0.002 | -0.865 |
| Dihydroergotamine | Serotonin agonist | 583.69 | + | 0.005 | -0.606 |
| Flunarizine | Calcium channel blocker | 404.51 | + | 0.009 | 1.363 |
| Lurasidone | Dopamine antagonist | 492.68 | + | 0.008 | -0.029 |
| Nizofenone | Neuroprotective | 412.87 | (+ + -) | 0.006 | -1.273 |
| Obidoxime | Cholinesterase reactivator | 288.31 | + | 0.001 | -0.844 |
| Penfluridol | Calcium channel blocker | 523.97 | + | 0.006 | 0.679 |
| Pipecuronium | Neuromuscular blocker | 602.90 | (+ +) | 0 | 0.306 |
| Pipenzolate | Cholinergic agonist | 354.47 | + | 0 | 0.231 |
| Rocuronium | Cholinergic agonist | 529.79 | + | 0.0002 | -0.062 |
| Siponimod | Sphingosine 1-phosphate receptor modulator | 516.61 | + | 0 | -0.126 |
| Tubocurarine | Cholinergic antagonist | 609.74 | (+ +) | 0.0003 | -0.769 |
| Vecuronium | Cholinergic antagonist | 557.84 | (+ +) | 0 | 0.328 |
| MW, molecular weight; a, charge at pH 7.4; b, solubility at pH 7.4 (mg/ml); | | | | | |

**Table S4**. Major therapeutic classes of screened 'neurology/psychiatry' charged candidates for cancer.

| Neuromodulator and Spasmolytics  (n=25) | apomorphine, l-dopa, benserazide, carbidopa, benztropine, piribedil, ropinirole, metixene, entacapone, amantadine, memantine, diphenhydramine, baclofen, biperiden, donepezil, rivastigmine, hyoscyamine, piperidolate, tizanidine, xylazine, tolperisone, rasagiline, safinamide, tranylcypromine, phenelzine. |
| --- | --- |
| Antipsychotic and anxiolytic (n=29) | aripiprazole, brexpiprazole, asenapine, blonanserin, clozapine, olanzapine, paliperidone, ziprasidone, risperidone, quetiapine, zotepine, sulpiride, baicalin, nicotine, promazine, chlorpromazine, fluphenazine, levomepromazine, perphenazine, trifluoperazine, triflupromazine, chlorprothixene, zuclopenthixol, flupentixol, fluspirilene, pimozide, haloperidol, spiperone, piperacetazine. |
| Antidepressants (n=23) | amoxapine, amitriptyline, clomipramine, desipramine, doxepin, imipramine, maprotiline, protriptyline, nortriptyline, opipramol, duloxetine, citalopram/ escitalopram, fluoxetine, fluvoxamine, paroxetine, sertraline, vilazodone, vortioxetine, trazodone, venlafaxine, oxitriptan, bupropion. |
| Anaesthetics (n=8) | bupivacaine, chloroprocaine, lidocaine, procaine, prilocaine, ropivacaine, tetracaine, hexylcaine. |
| Anti-inflammatory (with use in CNS symptoms) (n= 17) | aspirin, salicylic acid, diclofenac, gabapentin, ibuprofen, meclofenamic acid, nifulmic acid, parecoxib, tolfenamic acid, fosfosal, ketorolac, lornoxicam, loxoprofen, naproxen, zaltoprofen, nalbuphine, nefopam. |
| Antiseizures (n=2) | tiagabine, valproic acid. |
| Others | *Drugs from other category used in anxiety and stress*: propranolol, ademetionine, clonidine.  *Neuroprotective effect*: atorvastatin (lipid-lowering agent), tilorone (antiviral).  *Anti-hyperparathyroidism agent*: cinacalcet.  *Antihistamines (anti-migraine and motion sickness):* cinnarizine, meclizine, betahistine, hydroxyzine, ketotifen, nedocromil, promethazine, zolmitriptan, pizotifen.  *Anti-stroke and cerebral vasodilator*: dabigatran, ticlopidine, fasudil, ozagrel.  *Multiple sclerosis*: fingolimod, mitoxantrone, monomethyl fumarate.  *Smoke caseation*: varenicline, cytisine.  *Lactation suppression (acting on nervous system):* terguride, metergoline.  *Memory and cognition*: citicoline.  *Others*: l-arginine, l-glutamine, l-methionine, taurine, chlorogenic acid, glucosamine, rutin, atosiban, cholic acid. |

**Table S5**. Theoretical permeability values of repurposing antipsychotics of brain tumor

| Atypical Antipsychotics | | | | | |
| --- | --- | --- | --- | --- | --- |
|  | Drug Name | LogP* | LogD* | pKa* | LogBB* |
| 1 | Apipiprazole | 4.90 | 3.30 | 9.04-13.51 | -0.052 |
| 2 | Asenapine | 3.73 | 3.73 | 7.29* | 0.664 |
| 3 | Blonanserin | 5.67 | 4.27 | 8.79* | 0.403 |
| 4 | Brexpiprazole | 4.65 | 2.97 | 9.07-11.47 | 0.105 |
| 5 | Clozapine | 3.40 | 2.57 | 8.16* | 0.731 |
| 6 | Olanzapine | 3.38 | 2.56 | 8.16* | 0.376 |
| 7 | Paliperidone | 1.76 | 0.38 | 8.76-13.74 | -0.753 |
| 8 | Quetiapine | 2.81 | 2.29 | 7.76-15.12 | 0.028 |
| 9 | Risperidone | 2.63 | 1.25 | 8.76 | -0.064 |
| 10 | Sulpiride | 0.13 | -1.26 | 8.97-10.26 | -1.062 |
| 11 | Ziprasidone | 4.30 | 3.29 | 8.37-13.54 | 0.082 |
| 12 | Zotepine | 4.51 | 2.98 | 8.92 | 0.736 |
| Typical Antipsychotics | | | | | |
| 1 | Chlorpromazine | 4.54 | 2.74 | 9.20 | 0.066 |
| 2 | Chlorprothixene | 5.07 | 3.40 | 9.06 | 0.959 |
| 3 | Flupentixol | 4.50 | 3.68 | 8.15-15.59 | 0.597 |
| 4 | Fluphenazine | 3.97 | 2.73 | 8.61-15.59 | 0.594 |
| 5 | Fluspirilene | 5.78 | 4.35 | 8.81-11.99 | 0.160 |
| 6 | Haloperidol | 3.66 | 2.85 | 8.14-13.96 | -0.104 |
| 7 | Levomepromazine | 4.25 | 2.24 | 9.42 | 1.027 |
| 8 | Perphenazine | 3.69 | 2.45 | 8.61-15-59 | 0.619 |
| 9 | Pimozide | 5.83 | 4.33 | 8.88-13.1 | 0.249 |
| 10 | Piperacetazine | 3.63 | 1.72 | 9.32 | 0.008 |
| 11 | Promazine | 3.93 | 2.14 | 9.20 | 0.853 |
| 12 | Spiperone | 3.07 | 2.54 | 7.78-11.83 | 0.110 |
| 13 | Trifluoperazine | 4.66 | 3.36 | 8.68 | 0.875 |
| 14 | Triflupromazine | 4.81 | 3.01 | 9.20 | 0.821 |
| 15 | Zuclopenthixol | 4.22 | 3.41 | 8.15-15.99 | 0.621 |
| LogP, calculated from Chemicalize.com; pKa, strongest basic pKa-strongest acidic pKa; , logD value at pH 7.4, from chemicalize.com, logBB is from pKCSM, strongest acidic pKa. | | | | | |

**Table S6**. Highest clinical progress of each repurposed drugs for brain cancer treatment (till 13 March 2025).

| **Drug name** | **Clinical Trial** | **In vivo study (survival, reduction of tumour volume)** | **In vitro comparison between cancer and healthy cells** | **In vitro, cell viability, proliferation assay (direct evidence)** | **In vitro migration, mitochondrial activity inhibition, etc.** | **Target-based or in silico screening** |
| --- | --- | --- | --- | --- | --- | --- |
| Apomorphine |  |  |  | [1] |  |  |
| Amantadine |  | [2] |  |  |  |  |
| Amitriptyline |  |  |  |  | [3] |  |
| Amoxapine |  |  |  |  | [4] |  |
| Aripiprazole |  |  |  | [5] |  |  |
| Asenapine |  |  |  | [6] |  |  |
| Aspirin | Phase I [7] |  |  |  |  |  |
| Atorvastatin | Phase II [8] |  |  |  |  |  |
| Baclofen |  | [9] |  |  |  |  |
| Baicalin |  | [10] |  |  |  |  |
| Biperiden |  |  |  | [11] |  |  |
| Blonanserin |  |  |  | [12] |  |  |
| Brexpiprazole |  | [13] |  |  |  |  |
| Bupivacaine |  |  |  | [14] |  |  |
| Chlorogenic acid | Phase II [15] |  |  |  |  |  |
| Chloroprocaine |  |  |  | [16] |  |  |
| Chlorpromazine | Phase II [17] |  |  |  |  |  |
| Chlorprothixene |  |  |  | [18] |  |  |
| Cinacalcet |  | [19] |  |  |  |  |
| Cinnarizine |  |  |  |  | [20] |  |
| Citalopram |  |  | [21] |  |  |  |
| Clomipramine |  |  |  | [22] |  |  |
| Clozapine |  |  |  | [23] |  |  |
| Dabigatran |  |  |  |  | [24] |  |
| Desipramine |  |  |  |  | [25] |  |
| Diclofenac |  | [26] |  |  |  |  |
| Donepezil |  | [27] |  |  |  |  |
| Doxepin |  |  |  |  | [28] |  |
| Duloxetine |  | [29] |  |  |  |  |
| Escitalopram |  | [30] |  |  |  |  |
| Fasudil |  | [31] |  |  |  |  |
| Fingolimod | Early phase I  (NCT02490930) |  |  |  |  |  |
| Fluoxetine |  | [32] |  |  |  |  |
| Fluphenazine |  |  | [33] |  |  |  |
| Fluspirilene |  | [34] |  |  |  |  |
| Fluvoxamine |  | [35] |  |  |  |  |
| Gabapentin | Phase Ib/II [36] |  |  |  |  |  |
| Glucosamine |  |  |  | [37] |  |  |
| Haloperidol | Phase II (NCT06218524) |  |  |  |  |  |
| Ibuprofen |  | [38] |  |  |  |  |
| Imipramine | Phase II (NCT04863950) |  |  |  |  |  |
| Levomepromazine |  |  |  | [11] |  |  |
| Lidocaine |  | [39] |  |  |  |  |
| Maprotiline | Phase I [40] |  |  |  |  |  |
| Meclizine |  |  |  | [41] |  |  |
| Meclofenamic acid | Interventional pilot trial (NCT02429570) |  |  |  |  |  |
| Memantine | Phase Ib/II [36] |  |  |  |  |  |
| Monomethyl fumarate |  |  |  | [42] |  |  |
| Nicotine |  |  |  | [43] |  |  |
| Niflumic acid |  |  |  | [44] |  |  |
| Olanzapine |  | [45] |  |  |  |  |
| Paliperidone |  | [46] |  |  |  |  |
| Parecoxib |  |  |  | [47] |  |  |
| Paroxetine |  |  | [48] |  |  |  |
| Perphenazine |  | [48] |  |  |  |  |
| Pimavanserin |  | [49] |  |  |  |  |
| Pimozide |  | [50] |  |  |  |  |
| Prilocaine |  | [39] |  |  |  |  |
| Procaine |  | [39] |  |  |  |  |
| Propranolol | Phase I [7] |  |  |  |  |  |
| Protriptyline |  |  |  |  |  | [51] |
| Quetiapine |  | [52] |  |  |  |  |
| Rasagiline |  |  | [48] |  |  |  |
| Risperidone |  |  | [48] |  |  |  |
| Rivastigmine |  |  | [48] |  |  |  |
| Ropivacaine |  | [53] |  |  |  |  |
| Safinamide |  |  |  |  | [54] |  |
| Sertraline | Phase I/II [55] |  |  |  |  |  |
| Spiperone |  |  |  |  |  | [48] |
| Tacrine |  |  |  |  | [56] |  |
| Tetracaine |  |  |  | [57] |  |  |
| Tiagabine |  |  |  | [58] |  |  |
| Ticlopidine |  |  |  | [59] |  |  |
| Tolfenamic acid |  | [60] |  |  |  |  |
| Tranylcypromine |  |  |  | [61] |  |  |
| Trifluoperazine |  | [62] |  |  |  |  |
| Triflupromazine |  |  | [48] |  |  |  |
| Valproic acid | Phase II [63] |  |  |  |  |  |
| Varenicline |  |  | [48] |  |  |  |
| Vilazodone |  |  | [48] |  |  |  |
| Vortioxetine |  | [48] |  |  |  |  |
| Ziprasidone |  |  | [48] |  |  |  |
| Zolmitriptan |  |  | [48] |  |  |  |
| Zotepine |  |  | [48] |  |  |  |
| Note: The table is oriented in such a way that the furthest the blue colour boxes are to the left, the more clinical progress the drug made. Only the reference for highest clinical progress story is provided here.  References:  1. Lee, H., S. Kang, and W. Kim, *Drug Repositioning for Cancer Therapy Based on Large-Scale Drug-Induced Transcriptional Signatures.* PLoS ONE, 2016. **11**(3): p. e0150460.  2. Luo, Y.S., et al., *Amantadine against Glioma via ROS-Mediated Apoptosis and Autophagy Arrest.* Cell Death & Disease, 2024. **15**(11).  3. Bielecka-Wajdman, A.M., et al., *Reversing Glioma Malignancy: A New Look at the Role of Antidepressant Drugs As Adjuvant Therapy For Glioblastoma Multiforme*Cancer Chemotherapy and Pharmacology, 2017. **79**(6): p. 1249-1256.  4. Jing, Y., et al., *Therapeutic advantage of targeting lysosomal membrane integrity supported by lysophagy in malignant glioma.* Cancer Science, 2022. **113**(8): p. 2716-2726.  5. Kim, M.S., et al., *Src is the primary target of aripiprazole, an atypical antipsychotic drug, in its anti-tumor action.* Oncotarget, 2018. **9**(5): p. 5979-5992.  6. Soto Cerrato, V.K.M.M.G., Luis; Martinez Garcia, David; Perez Tomas, Ricardo Enrique; Soliva Soliva, Robert; Guallar Tasies, Victor; Diaz Bueno, Lucia; Quesada Pato, Roberto; Garcia Valverde, Maria, *Asenapine for Use in Cancer*, W.I.P. Organization, Editor. 2022, Fundacio Institut d'Investigacio Biomedica de Bellvitge (IDIBELL), Spain; Universitat de Barcelona, Spain; Universidad de Burgos, Spain; Nostrum Biodiscovery, S.L., Spain.  7. O'Rawe, M., et al., *Treatment of glioblastoma with re-purposed renin-angiotensin system modulators: Results of a phase I clinical trial.* Journal of Clinical Neuroscience, 2022. **95**: p. 48-54.  8. Altwairgi, A., et al., *Phase II Study of Atorvastatin in Combination with Radiotherapy and Temozolomide in Patients with Glioblastoma (ART): Final Analysis Report.* Annals of Oncology, 2019. **30**: p. ix20.  9. Zhu, L., et al., *Mechanism of Baclofen Inhibiting the Proliferation and Metastasis of GBM by Regulating YAP.* Journal of Chemistry, 2021. **2021**.  10. Hu, Y.-z., et al., *Antitumor effect of baicalin on rat brain glioma.* Chinese Journal of Oncology, 2013: p. 11-16.  11. Doello, K., et al., *Antitumor Effect of Traditional Drugs for Neurological Disorders: Preliminary Studies in Neural Tumor Cell Lines.* Neurotoxicity Research, 2022. **40**(6): p. 1645-1652.  12. Tsuchiya, N., et al., *Effect of Blonanserin on the Proliferation and Migration of Glioblastoma Cells.* Pharmazie, 2023. **78**(5): p. 37-41.  13. Suzuki, S., et al., *In vitro and in vivo anti-tumor effects of brexpiprazole, a newly-developed serotonin-dopamine activity modulator with an improved safety profile.* Oncotarget, 2019. **10**(37): p. 3547-3558.  14. Malet, A., et al., *The Comparative Cytotoxic Effects of Different Local Anesthetics on a Human Neuroblastoma Cell Line.* Anesthesia and Analgesia, 2015. **120**(3): p. 589-596.  15. Li, R., et al., *Cancer Differentiation Inducer Chlorogenic Acid Suppresses PD-L1 Expression and Boosts Antitumor Immunity of PD-1 Antibody.* International Journal of Biological Sciences, 2024. **20**(1): p. 61-77.  16. Perez-Castro, R., et al., *Cytotoxicity of Local Anesthetics in Human Neuronal Cells.* Anesthesia and Analgesia, 2009. **108**(3): p. 997-1007.  17. Pace, A., et al., *Efficacy and Safety of Chlorpromazine as an Adjuvant Therapy for Glioblastoma in Patients with Unmethylated Gene Promoter: RACTAC, a Phase II Multicenter Trial.* Frontiers in Oncology, 2023. **13**.  18. Kurita, J.I., et al., *Sertraline, chlorprothixene, and chlorpromazine characteristically interact with the REST-binding site of the corepressor mSin3, showing medulloblastoma cell growth inhibitory activities.* Scientific Reports, 2018. **8**(1): p. 13763.  19. Rodriguez-Hernandez, C.J., et al., *Cinacalcet inhibits neuroblastoma tumor growth and upregulates cancer-testis antigens.* Oncotarget, 2016. **7**(13): p. 16112-29.  20. Mena, M.A., et al., *Effects of calcium antagonists on the dopamine system.* Clinical Neuropharmacology, 1995. **18**(5): p. 410-426.  21. Sakka, L., et al., *Assessment of citalopram and escitalopram on neuroblastoma cell lines: Cell toxicity and gene modulation.* Oncotarget, 2017. **8**(26): p. 42789-42807.  22. Parker, K.A., et al., *The effect of tricyclic antidepressants on cutaneous melanoma cell lines and primary cell cultures.* Anti-Cancer Drugs, 2012. **23**(1): p. 65-69.  23. Karbownik, M.S., et al., *Antipsychotic Drugs Differentially Affect mRNA Expression of Genes Encoding the Neuregulin 1-Downstream ErbB4-PI3K Pathway.* Pharmacology, 2016. **98**(1-2): p. 4-12.  24. Vianello, F., et al., *Dabigatran antagonizes growth, cell-cycle progression, migration, and endothelial tube formation induced by thrombin in breast and glioblastoma cell lines.* Cancer Medicine, 2016. **5**(10): p. 2886-2898.  25. Ma, J., et al., *Antidepressant Desipramine Leads to C6 Glioma Cell Autophagy: Implication for the Adjuvant Therapy of Cancer.* Anti-Cancer Agents in Medicinal Chemistry, 2013. **13**(2): p. 254-260.  26. Johnsen, J.I., et al., *Cyclooxygenase-2 is expressed in neuroblastoma, and nonsteroidal anti-inflammatory drugs induce apoptosis and inhibit tumor growth in vivo.* Cancer Research, 2004. **64**(20): p. 7210-7215.  27. Mégalizzi, V., et al., *Screening of anti-glioma effects induced by sigma-1 receptor ligands: Potential new use for old anti-psychiatric medicines.* European Journal of Cancer, 2009. **45**(16): p. 2893-2905.  28. Higgins, S.C. and G.J. Pelkington, *The In Vitro Effects of Tricyclic Dugs and Dexamethasone on Cellular Respiration of Malignant Glioma.* Anticancer Research, 2010. **30**(2): p. 391-397.  29. Gao, H., et al., *S100B suppression alters polarization of infiltrating myeloid-derived cells in gliomas and inhibits tumor growth.* Cancer Letters, 2018. **439**: p. 91-100.  30. Chen, V.C.H., et al., *Escitalopram Oxalate Induces Apoptosis in U-87MG Cells and Autophagy in GBM8401 Cells.* Journal of Cellular and Molecular Medicine, 2018. **22**(2): p. 1167-1178.  31. Deng, L., et al., *Rho-kinase inhibitor, fasudil, suppresses glioblastoma cell line progression in vitro and in vivo.* Cancer Biology & Therapy, 2010. **9**(11): p. 875-84.  32. Liu, K.H., et al., *Fluoxetine, an antidepressant, suppresses glioblastoma by evoking AMPAR-mediated calcium-dependent apoptosis.* Oncotarget, 2015. **6**(7): p. 5088-5101.  33. Gil-Ad, I., et al., *Characterization of phenothiazine-incluced apoptosis in neuroblastoma and glioma cell lines: Clinical relevance and possible application for brain-derived tumors.* Journal of Molecular Neuroscience, 2004. **22**(3): p. 189-198.  34. Dong, Y., et al., *Identification of antipsychotic drug fluspirilene as a potential anti-glioma stem cell drug.* Oncotarget, 2017. **8**(67): p. 111728-111741.  35. Hayashi, K., et al., *Fluvoxamine, an anti-depressant, inhibits human glioblastoma invasion by disrupting actin polymerization.* Scientific Reports, 2016. **6**.  36. Mastall, M., et al., *A phase Ib/II randomized, open-label drug repurposing trial of glutamate signaling inhibitors in combination with chemoradiotherapy in patients with newly diagnosed glioblastoma: the GLUGLIO trial protocol.* BMC Cancer, 2024. **24**(1): p. 82.  37. Friedman, S.J. and P. Skehan, *Membrane-active drugs potentiate the killing of tumor cells by D-glucosamine.* Proceedings of the National Academy of Sciences, 1980. **77**(2): p. 1172-1176.  38. Gao, X., et al., *Ibuprofen induces ferroptosis of glioblastoma cells via downregulation of nuclear factor erythroid 2-related factor 2 signaling pathway.* Anticancer Drugs, 2020. **31**(1): p. 27-34.  39. Fan, X., et al., *Local anesthetics impair the growth and self-renewal of glioblastoma stem cells by inhibiting ZDHHC15-mediated GP130 palmitoylation.* Stem Cell Research & Therapy, 2021. **12**(1).  40. Petrosyan, E., et al., *Repurposing autophagy regulators in brain tumors.* International Journal of Cancer, 2022. **151**(2): p. 167-180.  41. Sandoval, J.A., et al., *Novel mtorc1 inhibitors kill glioblastoma stem cells.* Pharmaceuticals, 2020. **13**(12): p. 1-18.  42. Kumar, M., et al., *Lysine-Based C(60)-Fullerene Nanoconjugates for Monomethyl Fumarate Delivery: A Novel Nanomedicine for Brain Cancer Cells.* ACS Biomaterials Science & Engineering, 2018. **4**(6): p. 2134-2142.  43. Yamamura, M., et al., *Calcium mobilization during nicotine-induced cell death in human glioma and glioblastoma cell lines.* Anticancer Research, 1998. **18**(4A): p. 2499-2502.  44. Cui, W.L., et al., *Niflumic acid inhibits the proliferation, migration and invasion of glioma U87 cells.* Chinese Journal of Pathophysiology, 2020. **36**(10): p. 1887-1891.  45. Wang, Y.X., et al., *Autophagy Involvement in Olanzapine-mediated Cytotoxic Effects in Human Glioma Cells.* Asian Pacific Journal of Cancer Prevention, 2014. **15**(19): p. 8107-13.  46. Liu, Y.S., et al., *Paliperidone Inhibits Glioblastoma Growth in Mouse Brain Tumor Model and Reduces PD-L1 Expression.* Cancers, 2021. **13**(17).  47. Li, L.Y., et al., *Parecoxib inhibits glioblastoma cell proliferation, migration and invasion by upregulating miRNA-29c.* Biology Open, 2017. **6**(3): p. 311-316.  48. Lee, S., et al., *High-throughput identification of repurposable neuroactive drugs with potent anti-glioblastoma activity.* Nature Medicine, 2024. **30**(11): p. 3196-3208.  49. Liu, Z.-z., et al., *Identification of Pimavanserin Tartrate as a Potent Ca2+-Calcineurin-NFAT Pathway Inhibitor for Glioblastoma Therapy.* Acta Pharmacologica Sinica, 2021. **42**(11): p. 1860-1874.  50. Zhong, Y., et al., *Combinatorial targeting of glutamine metabolism and lysosomal-based lipid metabolism effectively suppresses glioblastoma.* Cell Reports Medicine, 2024. **5**(9): p. 101706.  51. Lin, W.-Z., et al., *From GWAS to drug screening: repurposing antipsychotics for glioblastoma.* Journal of Translational Medicine, 2022. **20**(1).  52. Wang, Y., et al., *Promoting oligodendroglial-oriented differentiation of glioma stem cell: a repurposing of quetiapine for the treatment of malignant glioma.* Oncotarget, 2017. **8**(23): p. 37511-37524.  53. Yin, D., et al., *Ropivacaine Inhibits Cell Proliferation, Migration and Invasion, Whereas Induces Oxidative Stress and Cell Apoptosis by circSCAF11/miR-145-5p Axis in Glioma.* Cancer Management and Research, 2020. **12**: p. 11145-11155.  54. Knez, D., et al., *Indoles and 1-(3-(benzyloxy) benzyl) piperazines: Reversible and selective monoamine oxidase B inhibitors identified by screening an in-house compound library.* Bioorganic Chemistry, 2022. **119**: p. 105581.  55. Halatsch, M.E., et al., *A phase Ib/IIa trial of 9 repurposed drugs combined with temozolomide for the treatment of recurrent glioblastoma: CUSP9v3.* Neuro-Oncology Advances, 2021. **3**(1): p. vdab075.  56. Zatta, P., et al., *Effects of tacrine upon murine neuroblastoma cells.* Journal of Neural Transmission, 1995. **102**(2): p. 113-123.  57. Mete, M., et al., *Neurotoxic effects of local anesthetics on the mouse neuroblastoma NB2a cell line.* Biotechnic & Histochemistry, 2015. **90**(3): p. 216-222.  58. Lee, C.-Y., et al., *The Effects of Antiepileptic Drugs on the Growth of Glioblastoma Cell Lines.* Journal of Neuro-Oncology, 2016. **127**(3): p. 445-453.  59. Lastakchi, S., M.K. Olaloko, and C. McConville, *A Potential New Treatment for High-Grade Glioma: A Study Assessing Repurposed Drug Combinations against Patient-Derived High-Grade Glioma Cells.* Cancers (Basel), 2022. **14**(11).  60. Eslin, D., et al., *Anticancer activity of tolfenamic acid in medulloblastoma: A preclinical study.* Tumor Biology, 2013. **34**(5): p. 2781-2789.  61. Sachkova, A., et al., *Combined applications of repurposed drugs and their detrimental effects on glioblastoma cells.* Anticancer Research, 2019. **39**(1): p. 207-214.  62. Bhat, K., et al., *The Dopamine Receptor Antagonist Trifluoperazine Prevents Phenotype Conversion and Improves Survival in Mouse Models of Glioblastoma.* Proceedings of the National Academy of Sciences, 2020. **117**(20): p. 11085-11096.  63. Krauze, A.V., et al., *A Phase 2 Study of Concurrent Radiation Therapy, Temozolomide, and the Histone Deacetylase Inhibitor Valproic Acid for Patients With Glioblastoma.* International Journal of Radiation Oncology, Biology, Physics, 2015. **92**(5): p. 986–992. | | | | | | |
